# Comprehensive circRNA Profiling Reveals a Testis-Specific circRNA circdmrt1 as a Novel Regulator of Gonadal Development in Crucian Carp

**DOI:** 10.3390/ijms27198593

**Published:** 2026-09-25

**Authors:** Dongmei Zhong, Faxiang Li, Ziyu Yang, Fang Gou, Yanmei Gao, Tian Bu, Qingfeng Liu, Yinggang Li, Wuhui Li, Qizhi Liu, Kaikun Luo, Rong Yang, Shaojun Liu

**Affiliations:** 1 Engineering Research Center of Polyploid Fish Reproduction and Breeding of the State Education Ministry, College of Life Sciences, Hunan Normal University, Changsha 410081, China; 202410140325@hunnu.edu.cn (D.Z.); 202220142856@hunnu.edu.cn (Z.Y.); 202470142961@hunnu.edu.cn (F.G.); 202320142793@hunnu.edu.cn (Y.G.); butian@hunnu.edu.cn (T.B.); liuqingfeng@hunnu.edu.cn (Q.L.); 202370142909@hunnu.edu.cn (Y.L.); liwuhui11@163.com (W.L.); lqz@hunnu.edu.cn (Q.L.); luokaikun@hunnu.edu.cn (K.L.); 2 Yuelushan Laboratory, Changsha 410128, China; 3 MOE Key Laboratory of Rare Pediatric Diseases, Center for Medical Genetics, School of Life Sciences, Central South University, Changsha 410081, China; lifaxiang@csu.edu.cn

**Keywords:** gonad development, circular RNAs, whole-transcriptome sequencing, miRNA

## Abstract

Fish gonad development is a central process in reproductive regulation, and elucidating its molecular mechanisms is highly important for developing aquatic genetic resources. However, the involvement of circular RNAs (circRNAs) in the gonad development of freshwater fish is unknown. In this study, a dynamic expression profile of circRNA during the development of red crucian carp testes was systematically constructed via whole-transcriptome sequencing. Notably, the circular characteristics and high testis-specific expression of the key circRNA circdmrt1, formed through the reverse splicing of the fourth exon of the *dmrt1* gene, have been experimentally validated. The overexpression of circdmrt1 promoted cell proliferation and inhibited the expression of apoptosis-related genes. Furthermore, in the hybrid offspring of red crucian carp and white crucian carp, circdmrt1 expression exhibited a pronounced paternal-line-biased pattern. Our data reveal that circdmrt1 may act as a ceRNA by sequestering miR-122-5p and thereby modulating DHRSX expression, leading to the activation of HEK-293T cell proliferation. This study reveals for the first time the regulatory features of testicular circRNAs in crucian carp, providing support evidence into the mechanisms of gonadal development in teleosts.

## 1. Introduction

Distant hybridization, as a core strategy for genetic innovation, promotes species diversification through genomic recombination and phenotypic remodeling, resulting in multilevel genetic effects. At the genomic level, allopolyploidy (such as the 4nAT lineage in cyprinids) occurs, breaking reproductive isolation [1]. Structurally, chromosomal rearrangements, including translocations and inversions, generate novel allelic combinations [2]. Phenotypically, this phenomenon manifests as significant hybrid vigor, characterized by increased growth rates and enhanced stress resistance [3,4,5]. Phenotypically, this phenomenon manifests as significant hybrid vigor, characterized by increased growth rates and enhanced stress resistance [6], representing a key bottleneck limiting breeding efficiency and necessitating the integration of artificial insemination and exogenous hormones (such as LHRH-a3) to overcome these limitations [7]. Red crucian carp (*Carassius auratus* red var., RCC), an ancient allopolyploid cyprinid, features a short generation cycle, strong reproductive capability, and genomic plasticity, along with a red coloration genetic marker [8], making it an ideal model for exploring the mechanisms of gonadal development in polyploid species and the reproductive regulatory networks involved in distant hybridization [9,10]. In RCC, successful distant hybridization has resulted in the generation of high-quality lines, such as allopolyploids (4nAT) from RCC (♀) and carp (♂) [11,12], riploid hybrids (3nRB) from RCC (♀) and *Pseudorasbora parva* (♂), and fertile diploid hybrids (4nRB) [13,14]. Using the hybrid offspring WR from the cross of white crucian carp (*Carassius cuvieri*, WCC, ♀) and RCC (♂), a new type of improved fish, WRII, was obtained by back-crossing the female hybrid (WR) with the male WCC. This hybridization improved both the growth and nutritional value of the fish’s muscle, representing an important genetic resource for the fishery economy [10,15,16]. Although the genomic integration patterns of these hybrid offspring provide unique insights into reproductive regulatory networks, the molecular mechanisms underlying gonadal development disorders in hybrids remain to be elucidated.

The development of gonads in teleosts is regulated by a combination of genetic and environmental factors. Sex-determining genes, such as *Sox9a*, can dominate gonadal differentiation through a cascade reaction [17]. *Dmrt1*, an evolutionarily conserved hub gene for sex regulation, establishes a bidirectional regulatory network by activating male pathway genes and inhibiting female factors [18,19,20]. In fish, the expression pattern of *Dmrt1* is species specific: in olive flounder, Z chromosome-linked *Dmrt1* maintains male development through a dosage effect [21]; the overexpression of *Dmrt1* in medaka can induce sex reversal in XX females [22]; and the loss of *Dmrt1* in zebrafish can lead to male infertility [23].

Circular RNA (circRNA), a type of noncoding RNA characterized by high stability and tissue specificity, typically forms closed-loop structures through back-splicing [24,25,26]. Recent studies have revealed that circRNAs act as novel regulatory molecules that participate in reproductive regulation through mechanisms that include competitive endogenous RNA (ceRNA) activity, protein interactions, and the encoding of short peptides. In mammals, ciRS-7 drives the differentiation of spermatogonial stem cells by adsorbing miR-7 [27], whereas circHIPK3 maintains the integrity of the Wnt/β-catenin signaling pathway to ensure the structure of seminiferous tubules [28]. Research in fish has indicated that for olive flounder, the testis-specific circRNA circdmrt1 activates male pathways by competitively binding cse-miR-196 [29], while circ-cacna1b in golden pompano influences the expression patterns of sex differentiation genes by regulating pma-miR-138b, highlighting the central role of the circRNA–miRNA–sex-determining factor axis in gonadal development [30]. Previous research on circRNAs in fish has focused mainly on model species, revealing, for example, an enrichment of sex-biased circRNAs in the Wnt/MAPK pathway in carp [31].

In addition to circRNAs, microRNAs (miRNAs) are another class of noncoding RNA molecules that are 18–26 nucleotides (nt) long. These molecules bind to the 3′ untranslated region (UTR) of target gene mRNAs through complementary base pairing, mediating post-transcriptional regulation through processes such as mRNA translation repression, nucleic acid cleavage, or poly(A) tail shortening. They are also widely involved in reproductive development [32,33]. for example, in zebrafish, miR-430 regulates the formation of reproductive ridges [34], while the identification of gonad-specific miRNAs (miR-129-3p/727-3p) in Nile tilapia revealed new mechanisms for sex dimorphism regulation [35]. Although there has been substantial research on gonadal development in fish, the post-transcriptional regulatory mechanisms involved in hybrid fish gonadal development, particularly the role of noncoding RNAs, remain unexplored.

In this study, we reveal a circRNA regulatory network involved in the testicular development of freshwater fish, including RCC, WCC, WR, and WRII. Through whole-transcriptome sequencing, a reference database of RCC circRNAs was constructed, enabling the identification of temporally differentially expressed circRNAs. We focused on the functional validation of circdmrt1 and elucidated its molecular mechanisms in regulating spermatogenesis, while additionally exploring the patterns of circdmrt1 in RCC × WCC hybrid strains (WR/WRII). Our study suggests that circdmrt1 can act as a ceRNA for miR-122-5p, facilitating the expression of DHRSX (dehydrogenase/reductase (SDR family) X-linked) and thereby modulating cell proliferation. Our findings provide valuable evidence and an experimental basis for future investigations into the molecular mechanisms of circular RNAs in crucian carp gonadal development.

## 2. Results

### 2.1. Profiling of CircRNAs in 4-Month-Old and 12-Month-Old Red Crucian Carp (RCC) Gonad Tissues

To explore the regulatory role and molecular mechanisms of circRNAs in the testicular development of red crucian carp, we extracted RNA from the testicular tissue of 4-month-old (immature stage, RCC_4 group) and 12-month-old (mature stage, RCC_12 group) RCC for whole-transcriptome sequencing. Finally, a ribo-depleted RNA-seq library (including circRNAs, mRNAs, and lncRNAs) and a small RNA library (including miRNAs) were constructed. We performed TopHat-Fusion nonlinear alignment on reads that did not align to the reference genome, revealing that in the RCC_12 group, the proportions of reads aligning to exonic, intronic, and intergenic regions were 85.15% ± 0.32%, 9.10% ± 0.15%, and 5.75% ± 0.09%, respectively. The corresponding proportions in the RCC_4 group were 91.71 ± 0.25%, 5.52 ± 0.11%, and 2.77 ± 0.07%, respectively (Supplementary Figure S1A, *p* < 0.01). These findings indicate an increase in transcript complexity during later stages of development, suggesting a significant increase in nonclassical splicing events in mature testicles. We identified a total of 16,742 circRNAs (total circRNAs), with the number of circRNAs in the RCC_12 group higher than the RCC_4 group. PCA analysis showed good intra-group reproducibility and significant inter-group differences (Supplementary Figure S1B). A total of 754 circRNAs were identified in both groups (Supplementary Figure S1C). The identified circRNAs were classified into three main categories on the basis of their origin: intronic type (ciRNA), exonic type (circRNA), and intergenic type (Figure 1A). Quantitative analysis revealed that exonic circRNAs accounted for approximately 88% of the genes in the RCC_12 group, which was greater than that in the RCC_4 group, whereas intergenic circRNAs accounted for approximately 19% of the genes in the RCC_4 group, which was greater than that in the RCC_12 group. Notably, the proportion of intronic ciRNAs was low in both groups, indicating that reverse splicing of exons is the main mechanism for circRNA generation in RCC testicles (Figure 1A). The expression distribution statistics revealed significant heterogeneity in the circRNAs in the RCC_12 group, with a wider range of expression abundance and a significantly increased proportion of outliers compared with those in the RCC_4 group (Figure 1B), suggesting that the dynamic expression of circRNAs is closely related to testicular maturation and may be involved in maintaining or degrading testicular function. Of note, testicular sexual maturation in crucian carp also involves an increased abundance of mature germ cells and the production of spermatozoa. Therefore, these differentially expressed circRNAs may also be closely associated with the generation of mature germ cells and spermatozoa in crucian carp. Statistics on the number of exons in the identified circRNAs revealed that circRNAs formed from three exons accounted for the greatest proportion (18.7%) of RCC testicles, followed by circRNAs formed from two exons (14.8%) and four exons (15.9%) (Figure 1C), suggesting that the number of exons is related to circularization efficiency or stability. As special splicing products of gene loci, multiple circRNAs can be produced from the same locus. Analysis of splicing complexity at gene loci revealed that 57.3% of the loci generated a single circRNA, 20.0% produced two circRNAs, and 22.7% generated three or more circRNAs (Figure 1D). CircRNAs can exert their biological functions by regulating their parental genes or independently of them, indicating that key genes may finely regulate testicular development through circular forms.

On the basis of differential expression analysis, this study revealed 1105 differentially expressed circRNAs (DEcircRNAs) in the testes of RCC, with 1003 significantly upregulated and 102 significantly downregulated circRNAs in the RCC_12 group (Figure 1E). Volcano plot visualization revealed that highly differentially expressed circRNAs were distributed mainly in the high-expression region (Figure 1F), suggesting that circRNAs have stage-specific regulatory characteristics during testis maturation, and importantly, the threshold was based on unadjusted *p* values, Subsequently, we will use qPCR and a series of additional validation methods to confirm the existence of the circRNA of interest.

Moreover, chromosome 7 (NC_039249.1) produced the most circRNAs (530) (Supplementary Figure S1D). The GO annotations of the host genes corresponding to DEcircRNAs cover three categories: biological process, cellular component, and molecular function (Supplementary Figure S2A). These annotations reveal these DEcircRNAs are predominantly derived from genes associated with testicular development and function. KEGG annotation of host genes corresponding to circRNAs in RCC testes revealed various metabolic and signaling pathways, with a focus on the top 20 enriched pathways, including the PI3K/Akt signaling and AMPK signaling pathways, which are involved in regulating metabolism and energy balance. Pathways such as oxidative phosphorylation and fatty acid elongation are linked to cellular energy metabolism, biosynthesis, and metabolic regulation, affecting membrane fluidity and the generation of signaling molecules (e.g., prostaglandins), which, in turn, influence the sperm acrosome reaction and cold tolerance (Supplementary Figure S2B). GO and KEGG pathway enrichment analyses of the host genes of the identified circRNAs indicate that they may participate in testicular development, functional maintenance, and response to environmental stress by regulating diverse metabolic pathways, signaling cascades, and cellular processes and suggest that circRNAs may exert analogous functions.

The prediction by IRESfinder and ORFfinder (v0.10.1) revealed that among the circRNAs in RCC testes, 14,302 circRNAs contained only ORFs, 547 circRNAs contained only IRESs, and 996 circRNAs contained both internal ribosome entry sites (IRESs) and ≥1 open reading frame (ORF) (Supplementary Figure S2C). These findings indicate that the differentially expressed circRNAs (DEcircRNAs) may possess coding potential in the testis of crucian carp.

### 2.2. Analysis of miRNAs During Testis Development in RCC

This study revealed 1715 mature miRNAs in the RCC testis, among which 504 miRNAs (29.39%) exhibited RCC_12 group-specific expression and 280 miRNAs (16.33%) were unique to the RCC_4 group. A total of 931 miRNAs (54.28%) were expressed during both periods, suggesting that stage-specific miRNAs may drive the molecular programs of specific developmental events (Figure 2A). Analysis of the nucleotide length distribution of the identified mature miRNAs revealed a typical distribution pattern for animal miRNAs: 22-nt miRNAs had the highest proportion (37.69%), followed by 23-nt (15.24%) and 21-nt (13.88%) miRNAs, whereas short-chain miRNAs of 18–20 nt had the lowest proportion (2.31% to 3.53%) (Figure 2B). Differential expression analysis of miRNA profiles during the testicular development process (from RCC_4 to RCC_12) (*p* < 0.05) revealed 441 significantly differentially expressed miRNAs, of which 109 miRNAs (24.72%) were upregulated, and 332 miRNAs (75.28%) were significantly downregulated (Figure 2C). The volcano plot revealed that the absolute values of the fold changes for the downregulated miRNAs were significantly greater than those for the upregulated miRNAs (Figure 2D). This expression polarity bias aligns with trends observed in mammalian spermatogenesis research, indicating that the reconstruction of post-transcriptional regulatory networks during the maturation stage may involve broad suppression of specific miRNAs to relieve their inhibitory effects on genes that initiate meiosis.

GO enrichment analysis revealed that the miRNA host genes are involved in the following functional categories: molecular function, characterized by DNA-binding transcription factor activity and the ability to bind specific DNA sequences; cellular component, components of the plasma and cytoskeleton membrane, suggesting that miRNAs participate in establishing and migrating the polarity of germ cells by regulating cytoskeletal reorganization (e.g., during sperm acrosome formation); and biological function, regulation of transcription, signal transduction, and multicellular organism development (Supplementary Figure S3A). KEGG enrichment analysis of the target genes of the significantly differentially expressed miRNAs revealed significant enrichment in pathways such as the MAPK signaling pathway, the TGF-β signaling pathway, the FoxO signaling pathway, and phosphoinositide metabolism, which can regulate spermatogonial stem cell self-renewal, the initiation of meiosis, and the clearance of abnormal cells (Supplementary Figure S3B).

### 2.3. Characterization of Circdmrt1 in RCC

By integrating expression abundance and host gene functional annotations, a key candidate circRNA, circdmrt1 (ID: circ6228), was selected (Supplementary Figure S4A). Its parent gene, *dmrt1* (double sex- and mab-3-related transcription factor 1 isoform X2, Gene ID: LOC113068729), is located on chromosome 5 (NC_039247.1) (Supplementary Figure S1C). GO enrichment analysis of the significantly differentially expressed circRNAs revealed that circdmrt1 has typical DNA-binding domains (GO:0003677) and transcription factor activity (GO:0003700) (Supplementary Figure S2A). Previous studies have shown that *dmrt1* is a highly conserved gene with high tissue specificity, is expressed predominantly in the testis, and plays a crucial role in sexual differentiation and male gonad development. Its circularization mechanism arises from the back-splicing of exon 4, flanked by long introns, resulting in a circular sequence 145 bp in length (Figure 3A).

To confirm the presence of circdmrt1, divergent primers and convergent primers were used to perform PCR amplification for cDNA and gDNA from 12-month-old RCC testes. The results revealed that the divergent primers amplified specific bands only in the cDNA, with no products in the genomic DNA (gDNA), confirming that the amplified circdmrt1 originated from cDNA rather than gDNA (Figure 3B). Amplification of the full-length circdmrt1 sequence using two pairs of back-splicing junction-spanning primers provided stronger evidence for its presence in the testis tissue of red crucian carp (Supplementary Figure S4B). To examine the different sensitivities of circRNA and linear RNA to RNase R digestion, we further validated their stability through an RNase R resistance experiment. The qRT-PCR results after RNase R digestion further confirmed the circular conformation of circdmrt1 (Figure 3C). These results suggest that circdmrt1 is stably present in crucian carp testicular tissues. We observed the development of the testis in RCC at different stages through HE staining and found that the testes of 4-month-old RCC were not yet mature and were primarily composed of spermatogonia, characterized by round or oval-shaped cells with a large nuclear–cytoplasmic ratio and larger supporting cell bodies. Spermatogonia begin to proliferate and form sperm sacs, and by 12 months of age, abundant mature sperm are visible in dense bundles within the lumen, which is consistent with the typical characteristics of the terminal stage of spermatogenesis in teleosts (Figure 3D). We then examined the expression of circdmrt1 in the testis at various stages and found that its abundance increased with testicular development, as also reflected by the transcriptome data (Figure 3E). These findings indicate that the dynamic expression of circdmrt1 may be related to the process of spermatogenesis. The expression levels of circdmrt1 in the liver, spleen, kidney, ovary, muscle, brain, intestine, pituitary gland, and testis of RCC were determined via qRT-PCR, which revealed significantly overexpression only in the testis (Figure 3F). To further investigate the mechanisms by which circdmrt1 functions during testicular development, we designed specific probes targeting circdmrt1. FISH revealed that circdmrt1 was predominantly localized in the cytoplasm of testicular cells (Figure 3G, Supplementary Figure S5A–E).

We noted that circdmrt1 was significantly upregulated during testicular development, which prompted us to investigate the function of circdmrt1. We observed that cell viability was significantly promoted by circdmrt1 overexpression in HEK-293T cells, as determined through a CCK-8 assay (Figure 3H). Additionally, an EdU incorporation assay revealed that the proliferation of HEK-293T cells was improved by the overexpression of circdmrt1 (Figure 3I). When we investigated the effects of circdmrt1 on apoptosis, we found that a high expression of circdmrt1 significantly increased the expression of the antiapoptotic gene *bcl-2* but inhibited the transcription of the proapoptotic gene *bax* (Figure 3J). These findings suggest that circdmrt1 promotes cell proliferation in HEK-293T cells, providing evidence supporting its potential functional role in crucian carp testicular development.

### 2.4. Circdmrt1 Formation Is Conserved in WCC, WR (WCC♀ × RCC♂), and WR-II (WR♀ × WCC♂)

During the production period, high-quality male RCCs were crossed with high-quality female WCCs to produce a hybrid offspring, WR. The female WR was subsequently backcrossed with the male WCC, resulting in the hybrid fish WRII (Figure 4A). We observed the development of the testis in WCC, WR, and WRII at different stages using HE staining. Our results revealed that the testes of 4-month-old fish had not yet matured and that the spermatogonia had begun to proliferate and form sperm sacs. By 12 months of age, abundant mature sperm were visible in dense bundles within the lumen at the terminal stage of spermatogenesis in teleosts (Figure 4B).

To verify its circular characteristics and interspecies conservation, specific primers spanning the splice sites were designed and used for RT-PCR amplification of cDNA from the testes of 12-month-old WCC, WR, and WRII. The products obtained were confirmed through Sanger sequencing and sequence alignment to have consistent base sequences and back-splicing sites (Figure 4C,D). Therefore, their circdmrt1 sequences are conserved. The qRT-PCR product circdmrt1 remained detectable after RNase R digestion, indicating the presence of circular RNAs in WCC, WR, and WRII (Figure 4E). To further study the expression characteristics of circdmrt1, we compared the expression of circdmrt1 in hybrid offspring (WR and WRII) and their respective parents and discovered that the expression abundance of circdmrt1 in male offspring was highly similar to that in the paternal line (Figure 4F,G), suggesting that its expression may be associated with paternal expression. In WRs and WRs, the expression of circdmrt1 was also strictly limited to the testis (Figure 4H), suggesting its potential functional conservation in male gonad development. β-actin was employed as a positive control for the in situ hybridization experiment, and a probe targeting the sense strand of circdmrt1 was designed as a negative control (Supplementary Figure S5A,B). FISH revealed that circdmrt1 was predominantly localized in the cytoplasm of testicular cells from WCC, WR, and WRII (Supplementary Figure S5C–E). Quantitative fluorescence analysis indicated that circdmrt1 is mainly localized in the cytoplasm of testis tissue, where it exerts its functional role (Supplementary Figure S5F). Collectively, these data suggest that circdmrt1 is highly conserved in different fish species and is associated with paternal expression.

### 2.5. Circdmrt1 RNA Physically Associates with mdo-miR-122-5p

Using whole-genome sequencing data, we determined that circdmrt1 has an ORF but lacks an IRES, indicating that it may generate functional peptide segments through nonclassical pathways (such as m^6^A modification-dependent translation mechanisms) or predominantly through noncoding regulation (Supplementary Figure S2C). Since many circRNAs function as miRNA “sponges” and circdmrt1 lacks an IRES, we tested whether circdmrt1 might bind and inhibit a specific miRNA.

Using a combined predictive strategy employing the miRNAda and miRanda algorithms, systematic screening was performed to identify potential interacting miRNAs of circdmrt1 in RCC testes, ultimately identifying six high-confidence target miRNAs. The expression of the top two miRNAs, cfa-miR-24_R-3 and mdo-miR-122-5p_R-4, significantly decreased from RCC_4 to RCC_12, and their expression patterns significantly negatively correlated with the continuous upregulation of circdmrt1, which is consistent with the characteristics of ceRNA regulatory mechanisms (Figure 5A). To confirm the prediction of the algorithms, we used qRT-PCR on 5-month-old and 12-month-old RCC testes to test the transcript levels of these two miRNAs. The results were consistent with the prediction (Figure 5B).

To confirm that miR-122-5p (mdo-miR-122-5p_R-4) binds to circdmrt1, luciferase reporter vectors containing the wild-type and mutated putative binding sites of circdmrt1 were constructed (Figure 5C). The luciferase activity of the circdmrt1 wild-type reporter was significantly lower when it was transfected with miR-122-5p mimics than when it was transfected with the control reporter or the mutated luciferase reporter (Figure 5D). However, miR-24 (cfa-miR-24_R-3) does not sponge circdmrt1 (Supplementary Figure S6A,B), and FISH colocalization further suggested that circdmrt1 may bind to miR-122-5p and exert its effects (Figure 5E). Argonaut 2 (Ago2) is the core component of the miRNA-induced silencing complex and is enriched in the cytoplasm. Here, Ago2-RNA immunoprecipitation (RIP) was performed to determine whether circdmrt1 serves as a platform for Ago2 and miR-122-5p assembly. The results revealed that circdmrt1 was specifically enriched in HEK-293T cells transfected with miR-122-5p mimics (Figure 5F). Next, we performed RIP with an Ago2 antibody in RCC testes and found that both circdmrt1 and miR-122-5p were significantly enriched (Figure 5G). These results support a model whereby circdmrt1 may serve as a binding platform for Ago2 and miR-122-5p, suggesting a potential sponge-like interaction for miR-122-5p in RCC testes.

### 2.6. Circdmrt1 Relieves the Repressive Effect of miR-122-5p on the Expression of DHRSX

We subsequently predicted the downstream target genes of miR-122-5p. In RCC, the predicted target genes of miR-122-5p included *gdf11*, *cog6*, *plgb*, *mpp1*, *dhrsx*, *vps25*, *kit20a*, and *plpp2* (Figure 6A). We tested the transcript levels of these eight targets in 5-month-old and 12-month-old RCC testes, with circdmrt1 serving as the control. We observed that *dhrsx* and *gdf11* were the most abundant validated genes (Figure 6B) and that there were many complementary base pairs between miR-122-5p and the 3’ untranslated region (3’ UTR) of the *gdf11* and *dhrsx* mRNAs, with *dhrsx* genes being more common (Figure 6C). Next, we performed RIP with an Ago2 antibody in RCC testes to identify the gene that interacts with circdmrt1. The results revealed that *dhrsx* was significantly enriched, but *gdf11* was not (Figure 6D). To investigate the regulatory interaction between circdmrt1 and *dhrsx*, we performed FISH analysis of circdmrt1 and immunodetection of DHRSX protein in RCC testes, which revealed that circdmrt1 predominantly colocalized with DHRSX in the cytoplasm of germ cells (Figure 6E). We subsequently investigated the interaction between circdmrt1, miR-122-5p, and DHRSX; colocalization experiments demonstrated that miR-122-5p and DHRSX were co-expressed in the cytoplasm of testicular cells (Figure 6F). Compared with the control, the transfection of miR-122-5p in HEK-293T cells significantly inhibited DHRSX protein expression, an effect that was reversed by the overexpression of circdmrt1 (Figure 6G). A subsequent dual-luciferase assay con-firmed that miR-122-5p directly bound to the 3’UTR of dhrsx, thereby inhibiting DHRSX protein expression (Figure 6H,I). These findings suggest that circdmrt1 and miR-122-5p likely regulate DHRSX at the post-transcriptional level. Thus, during testicular development in RCC, circdmrt1 may regulate DHRSX expression. Small interfering RNA (siRNA) molecules are double-stranded RNAs of approximately 20–25 nucleotides in length that mediate RNA interference (RNAi) by sequence-specific binding to complementary target mRNAs, leading to their degradation or translational repression. To investigate the physiological role of circdmrt1 in crucian carp, two independent siRNAs (si-circdmrt1-1 and si-circdmrt1-2) were designed to specifically target the BSJ of circdmrt1. The knockdown efficiency of each siRNA was first validated using qPCR in primary cultured crucian carp cells or HEK-293T cells (Figure 6J). Given that naked siRNA is susceptible to degradation by nucleases in vivo, we adopted a continuous intraperitoneal injection protocol to extend its duration of action: 10 μg of siRNA (dissolved in 200 μL sterile PBS) was administered to 6-month-old crucian carp on days 1, 2, and 3, with control fish receiving equal volumes of PBS (Figure 6K). Each experimental group consisted of six fish as independent biological replicates. On day 4, fish were anesthetized and dissected, and various tissues, including those of the testis, liver, brain, and heart, were collected. qPCR analysis revealed that both siRNAs significantly reduced circdmrt1 expression in the testis (*p* < 0.05), with the most pronounced effect observed in the testis, followed by the brain, while no notable changes were detected in the other tissues. Importantly, linear *dmrt1* mRNA levels were not significantly affected by either siRNA treatment (*p* > 0.05), confirming that the designed siRNAs specifically target the BSJ of circdmrt1 without affecting the expression of its host gene linear transcript. We further examined expression changes in the downstream target gene *dhrsx* and the apoptosis-related gene *bcl-2* upon circdmrt1 knockdown to assess its impact on downstream signaling pathways (Figure 6L,M, Supplementary Figure S7A,B). Based on our findings on circdmrt1 and miR-122-5p, we speculate that circdmrt1 may function as a ceRNA by sponging miR-122-5p and promoting DHRSX protein expression, influencing the expression of anti- and proapoptotic genes and thereby promoting cell proliferation.

## 3. Discussion

In this study, the dynamic expression characteristics of circRNAs during testicular development in RCC were systematically analyzed. A total of 16,742 circRNAs were identified, with 15,452 and 2044 circRNAs detected in the 12-month-old (RCC_12) and 4-month-old (RCC_4) testes, respectively. Under a more stringent filtering criterion requiring circRNA detection in at least two biological replicates, 10,516 circRNAs were retained in the RCC_12 group, whereas only 1269 circRNAs were retained in the RCC_4 group, indicating that this disparity is not merely driven by events detected in a single sample. Technical factors, including sequencing depth (approximately 10 Gb of clean data per sample), BSJ read abundance (circRNAs with ≥2 junction reads), and library complexity, appear insufficient to fully account for this discrepancy. We speculate that this difference may primarily reflect the complexity of testicular cellular composition and physiological status across developmental stages: the 12-month testes are fully mature and encompass a complete spectrum of spermatogenic cell types, whereas the 4-month testes are immature and lack late-stage germ cell populations. Given that many circRNAs exhibit cell-subtype-specific expression, the stage-restricted cellular milieu may profoundly influence the circRNA detection landscape. Nevertheless, the current dataset does not permit direct validation of this speculation. Future studies incorporating a broader range of developmental stages and increased biological replicates will be necessary to systematically characterize the dynamic changes and cellular origins of circRNAs during testicular development. Exon-type circRNAs dominated in both sample groups, with a greater proportion in the RCC_12 group, indicating that as the testes mature, the complexity of the transcriptome increases, potentially driving the splicing machinery to generate novel circRNA subtypes through nonclassical circularization. This results in fine-tuned post-transcriptional regulation of genes related to spermatogenesis. Analysis of the complexity of gene locus splicing revealed that 22.7% of gene loci could produce ≥3 circRNA isoforms. This phenomenon of multiple circularizations may coordinate the precision of testicular development through multilayered regulatory networks. For example, different circRNA isoforms produced from the same gene locus may form dynamic regulatory modules by competitively binding to miRNAs or regulating alternative splicing of target genes. However, these differentially expressed transcripts are associated with testicular maturation, although the extent to which they reflect cell composition changes versus cell-intrinsic regulatory events remains to be determined. Future research integrating single-cell transcriptomic approaches, such as scRNA-seq, would facilitate a more detailed resolution of the expression dynamics and regulatory networks of circdmrt1 and its associated genes in distinct testicular cell populations at the single cell level.

Differential expression analysis revealed 996 differential circRNAs that simultaneously carry an IRES and an ORF, suggesting that they may generate functional peptides through cap-independent translation. For example, circRNAs with complete IRES elements may directly participate in signal transduction, whereas circRNAs lacking an IRES (such as circdmrt1) tend to adsorb miRNAs through ceRNA mechanisms, indirectly regulating target gene expression. These findings reveal a dual-functional paradigm of circRNAs in the reproductive regulation of teleost fish, providing new perspectives for reproductive biology research: they can act both as noncoding RNAs that regulate gene networks and as coding short peptides that are directly involved in signaling pathways. GO and KEGG analyses of the parent genes of the differentially expressed circRNAs revealed significant modular characteristics, with these pathways highly consistent with studies on zebrafish testicular development [23,36]. However, the genomic characteristics of RCC may confer unique complexity to its regulatory networks. It is worth noting that in the present study, the analysis of DE circRNAs was performed using an unadjusted *p* < 0.05 threshold for screening, which may introduce a certain number of false-positive results. The identification and characterization of specific circRNAs of interest will require further experimental validation in subsequent studies. In the future, we will increase the sample size and employ FDR multiple-testing correction for screening to further improve the robustness of our analyses.

This study identified 1715 miRNAs, of which 441 were significantly differentially expressed, an amount that far exceeds the approximately 1200 miRNAs found in zebrafish testes and the 1423 miRNAs recorded in the cyprinid gonadal miRNA database. This may be related to the expansion of miRNA families caused by the specific genome of RCC. The downregulated miRNAs in the RCC_12 group were significantly enriched in pathways related to the maintenance of germ stem cells and hormonal responses, whereas the upregulated miRNAs were closely associated with sperm morphology development (such as flagellum assembly and acrosomal enzyme activation), suggesting that miRNAs have a temporal regulatory function during different stages of spermatogenesis. It is worth noting that in mammals, miR-122-5p has been confirmed to be involved in the regulation of spermatogenesis, promoting the proliferation of spermatogonial stem cells [37], though its function in fish still needs further verification.

Circdmrt1 is formed through the back-splicing of exon 4 of the dmrt1 gene. Its circular characteristics have been confirmed through RT-PCR, Sanger sequencing, and RNase R resistance experiments, and it is highly expressed in the testis, suggesting its functional conservation in male gonad development. Tissue specificity analysis revealed that the expression level of circdmrt1 in the RCC_12 group was significantly upregulated, suggesting that circdmrt1 may be involved in biological processes associated with gonadal development, germ cell abundance, and spermatogenesis in crucian carp. However, these correlative observations warrant further functional validation. Here, we demonstrated that circdmrt1 can serve as a modulator of cell growth in HEK-293T cells. Interestingly, we found that circdmrt1 significantly affected cell proliferation. To further explore the specific mechanisms by which circdmrt1 functions in testicular development, determining its subcellular localization is necessary. If a circRNA is localized in the nucleus, it may regulate transcription or epigenetic modifications; if it is localized in the cytoplasm, it is more likely to function as a miRNA sponge or a protein interaction molecule or participate in protein translation. Therefore, we conducted FISH experiments and confirmed that circdmrt1 is localized in the cytoplasm. Given its lack of IRES characteristics, it may adsorb miRNA miR-122-5p through a ceRNA mechanism, thereby alleviating the inhibition of target genes by miRNAs and regulating gonadal homeostasis.

*Dmrt1* is recognized as a key gene for gonadal development that is highly expressed in the testis. Similarly, our results showed that circdmrt1 expression levels in the hybrid fish WR and WRII were highly similar to those of the paternal line. This suggests that circdmrt1 may mitigate the disruptive effects of hybridization on male gene expression, thereby supporting normal testicular development. However, further experimental work and larger sample sizes are still needed to validate these findings.

We speculate that circdmrt1 may participate in the regulation of testicular development via the miRNA–circRNA–mRNA pathway, enhancing the activity of downstream target gene promoters. We predicted the proteins that bind to miR-122-5p through software analysis and found through qPCR that the expression of the DHRSX protein significantly changed during testicular development in red crucian carp. Research on the dhrsx gene, located in the pseudoautosomal regions of the human X and Y chromosomes, has focused primarily on its roles in biological growth, tissue formation, and other processes [38]. Furthermore, studies have identified *dhrsx* as a critical candidate gene for azoospermia treatment [39]. The interaction between *dhrsx* and miR-122-5p was confirmed by constructing a luciferase overexpression vector and conducting a dual-luciferase reporter assay. Through Ago2 RIP and protein interaction FISH, the regulatory network of circdmrt1 (circdmrt1/miR-122-5p/DHRSX) was elucidated, and its function in spermatogenesis was systematically analyzed. Based on the findings described herein, we speculate that circdmrt1 may participate in the regulation of crucian carp testicular development through a ceRNA network (Figure 7), although this hypothesis requires further experimental validation. It should be noted that this working model is derived from Ago2-RIP, FISH colocalization, and dual-luciferase reporter assays, and it remains to be determined whether the endogenous abundance of circdmrt1 is sufficient to effectively sequester miR-122-5p under physiological conditions. Future studies will therefore aim to quantify the absolute copy numbers of circdmrt1 and miR-122-5p using absolute quantitative PCR (ddPCR) and analyze the stoichiometric relationship among miR-122-5p, circdmrt1, and DHRSX, thereby allowing a more rigorous assessment of the proposed ceRNA model.

Here, the dynamic characteristics of circRNAs in the testicular development of RCC and the genetic regulatory patterns of circdmrt1 were systematically analyzed. However, the cell-based functional assays (CCK-8, EdU, and transfection experiments) in this study were all performed in HEK-293T cells, which are widely used as a heterologous expression system for cell biology studies due to their high transfection efficiency and stable experimental conditions, making them suitable for preliminary validation of the circdmrt1/miR-122-5p/DHRSX regulatory mechanism. However, HEK-293T cells are derived from human embryonic kidney and are not a fish testicular cell line; thus, the intracellular environment differs substantially from that of crucian carp gonadal tissue. Therefore, the functional data obtained in HEK-293T cells in this study serve only as supportive mechanistic evidence, providing clues and a foundation for future functional studies in fish in vivo or in fish-derived cell lines, and should not be directly equated with the physiological functions of circdmrt1 in crucian carp testicular development. Future work will be dedicated to the establishment of primary cell lines from crucian carp and the further validation of the mechanistic findings. Then, the results of the whole transcriptome and qPCR-based basic validation experiments were obtained from three biological replicates, which is considered the minimum acceptable number of replicates. Thus, in the future, we still need to increase the sample size to improve the reliability of our results. In the present study, we have shown that in vivo knockdown of circdmrt1 affects the expression of downstream dhrsx and apoptosis-related genes, providing direct evidence for the role of circdmrt1 in promoting testicular development in crucian carp. However, to ensure the completeness of mechanistic validation, our future efforts will focus on rescue experiments combining circdmrt1 knockdown by shRNA, and with miR-122-5p inhibition, coupled with histological examination of testicular development. In addition, we performed in vivo knockdown experiments using two siRNAs with distinct sequences and obtained similar results, which largely ruled out the possibility of false-positive results due to off-target effects from a single siRNA; we recognize that a scrambled siRNA control would further strengthen the rigor of our conclusions. Therefore, in future studies, we will include a scrambled siRNA control group to further exclude nonspecific effects.

## 4. Materials and Methods

### 4.1. Samples

The fishes used in this study were cultured at the Engineering Research Center of Polyploid Fish Breeding and Reproduction of the State Education Ministry, located at Hunan Normal University. Starting from the day of hatching, testis samples (red crucian carp, white crucian carp, WR (WCC ♀ × RCC ♂), WR-II (WR ♀ × WCC ♂), through distant hybridization) were collected at different months. Sex was preliminarily determined by morphological observation of gonads upon dissection; testes appeared as milky-white, elongated structures. In cases where the sex could not be distinguished and the gonadal location was not yet identifiable in juvenile fish (e.g., 2 months of age), testicular tissues were collected from the celomic cavity region near the kidney and further confirmed by histological examination using H&E staining. Tissues were collected from crucian carp at different ages (2, 4, 6, 8, 10, and 12 month), six individual fish per group, snap frozen in liquid nitrogen, stored at −80 °C, and three individuals were randomly selected from the frozen samples collected in 4-month and 12-month, respectively, as the three biological replicates for whole-transcriptome sequencing, qPCR, FISH, and other histological analyses, with each biological replicate corresponding to a single fish. Throughout the rearing period, all fish were maintained under uniform conditions, including stable water temperature (28 ± 1 °C) and standardized feeding protocols (3% of body weight per day), to minimize any confounding effects of environmental factors on fish development.

### 4.2. Whole-Transcriptome Sequencing Analysis and circRNA Identification

The whole-transcriptome sequencing assay was provided by LC Sciences. In this study, circRNA sequencing analysis was performed on testicular tissue from RCC. Total RNA was extracted from the testis tissue of each fish using TRIzol reagent (Invitrogen, Carlsbad, CA, USA). A dual library construction strategy was employed, combining ribosomal RNA removal (rRNA-) with RNase R linear RNA digestion to enrich circRNAs and suppress linear RNA interference. Two experimental conditions, 4 months of age (RCC_4, pre-maturation) and 12 months of age (RCC_12, maturation stage), were set up (each with three biological replicates). Sequencing was completed using an Illumina No-vaSeq™ 6000 (LC-Bio Technology Co., Ltd., Hangzhou, China) sequencer, and the data (mRNA, lncRNA, and circRNA information) were obtained with a sequencing depth of approximately 10 Gb of clean data per sample. After quality control and adapter filtering of the raw data with FASTQC (v0.10.1) and Cutadapt (v1.10), the effective data proportion was 95.48–96.11%, with Q20 values reaching 98.46–94.04% and Q30 values reaching 91.81–94.80%. The reads were aligned to the NCBI reference genome (GCA_003368295.1) using TopHat (v2.0.4), and unmapped reads were mapped to the reference genome using TopHat-Fusion (2.1.0). CircRNAs were identified via back-splice junctions (BSJs). CircRNAs were further identified using the combined algorithms CIRCexplorer2 (v2.2.6) and CIRI (v2.0.2), which employ a back-splice junction recognition strategy to filter nonmapped reads [40], a maximum genomic distance between splice sites ≤ 100 kb, and a minimum read length coverage of 20 bp. The result from the two algorithms were then integrated to enhance the reliability of circRNA identification. CircRNA expression levels were normalized using SRPBM (splice reads per billion mapped reads). Differential expression analysis was performed via edgeR (v3.22.5) [41] with the parameters set to unadjusted *p* < 0.05 and a fold change >2 or <0.5. CircRNAs with a *p* value less than 0.05 and a differential fold change greater than 2 were classified as differentially expressed (DE) circRNAs (both conditions must be met). For functional annotation and pathway enrichment analysis, the host genes corresponding to DE circRNAs were mapped to the Gene Ontology (GO) and Kyoto Encyclopedia of Genes and Genomes (KEGG) databases. Enrichment analysis was performed using the OmicStudio Universal Enrichment Analysis platform developed by LC-Bio.

### 4.3. Identification of miRNAs

Total RNA extracted from the testicular tissues was collected from different individuals of the same population of 4-month-old and 12-month-old red crucian carp (N = 3/group) and was used to construct libraries with TruSeq Small RNA Sample Prep Kits (Illumina, San Diego, CA, USA). Library quality was assessed using the Agilent 2100 Bioanalyzer (Agilent, CA, USA), which revealed that the fragment peaks were concentrated between 145 and 160 bp, meeting the standards for miRNA libraries. Single-end 50 bp (SE50) sequencing was subsequently performed on the Illumina HiSeq 2500 platform, yielding an average of 12.5 ± 1.3 million raw reads per sample. Quality control was conducted via FastQC (v0.11.9), and low-quality sequences (Phred score < 20), adapter contamination, and unusually short or long fragments (<15 nt or >35 nt) were removed via Cutadapt (v1.18), resulting in high-quality clean data with an average retention rate of 82.4%.

The sequences were then aligned to the Rfam (v14.2) and Repbase (v21.12) databases via Bowtie2 (v2.3.5), and nontargeted RNAs such as rRNA (12.7%), tRNA (7.3%), small nucleolar RNA (snoRNA) (3.1%), and transposable elements (5.9%) were removed to ensure the specificity of the miRNA annotations. Furthermore, pre-miRNA hairpin structures were predicted using miRDeep2 (v2.0.1) in conjunction with the RCC genome (CAB_v2.0). Classical Dicer cleavage sites and isoform variations were identified, and expression levels were quantified using the transcripts per million (TPM) normalization method. Only uniquely mapped reads with perfect matches to the reference miRNA sequences were retained for further analysis. The average mapping rate was above 85%.

Differential expression analysis of miRNAs was performed by LC-Bio. Raw miRNA read counts were normalized according to the method reported by Li et al. [42]. Specifically, sequences shared by all libraries were extracted for normalization using a median-based linear regression correction strategy. Briefly, a reference dataset was built from the median copy numbers of these shared sequences across all samples. Log-transformed copy numbers of samples and the reference dataset were calculated, and stably expressed sequences with moderate variation between each sample and the reference were screened out. A linear regression model was fitted on these stable sequences between each sample and the reference dataset to derive sample-specific correction factors. Raw copy numbers were multiplied by the corresponding correction factor of each sample to generate normalized copy numbers, and the normalized expression matrix was constructed accordingly. MiRNAs with normalized copy number ≥10 in at least one group were retained for subsequent statistical analysis. A value of 0.01 was imputed for samples with zero read counts to prevent errors in logarithmic calculation. As biological replicates were available for the two-group comparison, a two-tailed unpaired Student’s *t*-test with unequal variances was applied. MiRNAs satisfying both criteria of unadjusted *p* < 0.05 and |log2(fold change)| > 2 were identified as differentially expressed miRNAs.

### 4.4. Construction of the ceRNA Network

Differentially expressed circRNAs were selected as candidate molecules for con-structing the ceRNA regulatory network. TargetScan (5.0) and miRanda (3.3a) were used to predict the miRNAs potentially interacting with these circRNAs (a binding free energy ≤−20 kcal/mol). To ensure the reliability of the predictions, only circRNA–miRNA interaction pairs identified by both tools were retained. Subsequently, TargetScan and miRanda were again used to predict the downstream target mRNAs of the identified miRNAs. Similarly, only miRNA–mRNA interaction pairs predicted by at least two databases and present within the differentially expressed mRNA dataset were kept. Finally, the filtered circRNA–miRNA and miRNA–mRNA interaction pairs were integrated. Visualization of the resulting network was performed using the OmicStudio cloud platform provided by LC-Bio.

### 4.5. Target Gene Prediction

By searching circRNA sequences and regions with base complementary pairing to miRNAs via TargetScan (5.0) software (score percentile > 50) [43] a and selecting targets with lower energy stable binding sites (free energy <−10 kcal/mol) via miRanda(3.3a) [44], reliable circRNA-miRNA interactions were identified.

### 4.6. Validation of circdmrt1 by RT-PCR

Total RNA was extracted from the testes of four fish species (RCC, WCC, WR, and WRII) during sexual maturity. cDNA was synthesized via the HiScript II 1st Strand cDNA Synthesis Kit (Vazyme, Nanjing, China). Specific primers for circRNAs were designed, comprising two pairs: one pair of divergent primers aimed outward to amplify the circRNA containing the back-splice junction and another pair designed to amplify the linear mRNA form of the fourth exon of the *dmrt1* gene (convergent primers, as control). The genomic DNA (gDNA) used in the experiment was obtained from the tail vein blood of the four fish species during sexual maturity, with ACD blood anticoagulant (Sangon Biotech, Shanghai, China) added to prevent clotting, and extracted using the TIANamp Genomic DNA Kit (Tiangen, Beijing, China). The expression was used as a control for the specificity of the divergent primers. The conditions for RT-PCR amplification were as follows: 95 °C for 3 min, followed by 35 cycles of 95 °C for 15 s, 59 °C for 15 s, and 72 °C for 30 s, with a final extension at 72 °C for 5 min.

After RT-PCR amplification of cDNA from the four fish species via Phanta SE Super-Fidelity DNA Polymerase (Thermo Fisher Scientific, Waltham, MA, USA), the products were separated via 2.0% agarose gel electrophoresis. The target PCR products were then purified using the FastPure Gel DNA Extraction Mini Kit (Vazyme, Nanjing, China).

### 4.7. RNase R Treatment

RNA from the testes of sexually mature red crucian carp was treated with RNase R (Lucigen, RNR07250, Middleton, WI, USA) at a concentration of 5 U/μg on ice, and enzyme-free water was used as a control. The samples were incubated at 37 °C for 30 min, and the enzyme activity was terminated by heating at 70 °C for 10 min. The treated RNA was then subjected to quantitative analysis via qRT-PCR. Samples without RNase R treatment served as the negative control, and *dmrt1* mRNA expression was used as a positive control to assess the efficiency of RNase R digestion.

### 4.8. Real-Time qPCR

RCC, WCC, WR, and WRII at 4 months and 12 months of age were maintained under natural conditions in a shared pond at the Aquaculture Experimental Base of Hunan Normal University. Gonadal, brain, and liver tissues were rapidly dissected following anesthesia, immediately snap-frozen in liquid nitrogen, and stored at −80 °C until further processing. RNA concentration and purity were measured using a NanoDrop 2000 spectrophotometer (Thermo Fisher Scientific, Waltham, MA, USA) (A260/A280 ratio between 1.8 and 2.0). cDNA was synthesized using the PrimeScript™ RT Reagent Kit with gDNA Eraser (Takara, Kusatsu, Japan) according to the manufacturer’s instructions. Briefly, 1 μg of total RNA was treated with gDNA Eraser to remove genomic DNA contamination, followed by reverse transcription using random hexamers and oligo(dT) primers. No-RT controls were included to verify the absence of genomic DNA contamination.

Divergent primers were designed to specifically amplify circdmrt1 across the back-splice junction site. The primer sequences are provided in Table S1. β-actin and 18S rRNA were used as the endogenous reference genes. Additionally, the geometric mean of both reference genes was used to calculate the relative expression levels. RT-qPCR was performed using PowerUp SYBR Green Master Mix (Applied Biosystems, Waltham, MA, USA) on a QuantStudio 5 system (Life Technologies, Carlsbad, CA, USA). Each 20 μL reaction mixture contained 10 μL of 2× SYBR Green Mix, 0.4 μM of each primer, 2 μL of diluted cDNA, and nuclease-free water. The thermal cycling protocol was as follows: 95 °C for 30 s, 40 cycles of 95 °C for 5 s, 60 °C for 30 s, and 72 °C for 30 s, followed by melt curve analysis. No-template controls (NTC) and no-reverse-transcriptase controls (NRT) were included to monitor contamination and genomic DNA residue.

### 4.9. Paraffin Embedding and Sectioning

Fresh testis samples from four fish species were fixed in RNA-free 4% paraformaldehyde (Servicebio, Wuhan, China) at room temperature for 24 h. After fixation, the samples were washed with RNA-free sterile water for 20 min. They were then subjected to a gradient dehydration process: treatment with 75% and 85% absolute ethanol for 2 h, followed by 95% absolute ethanol for 1 h, and finally xylene for 1 h to achieve transparency. The samples were then placed in embedding molds and filled with wax to solidify the material. Once embedded, the wax blocks were sliced into 3-µm-thick sections, placed on slides coated with protein glycerol after being soaked in poly-L-lysine, and then dried overnight in an oven at 42 °C.

### 4.10. Hematoxylin-Eosin (HE) Staining

After xylene immersion for deparaffinization, the tissue sections were rehydrated via a gradient of absolute ethanol. Following an 8-min treatment with hematoxylin, the sections were washed with water, then acidified and alkalized. The samples were then dehydrated via a gradient of absolute ethanol and xylene, stained with eosin, and finally coverslipped with mounting medium.

### 4.11. RNA Fluorescence In Situ Hybridization (FISH)

We designed a specific probe (20 nt in length) covering the BSJ region and 10 nt of flanking sequences on each side to ensure hybridization specificity. This was synthesized by Sangon (Shanghai, China) and labeled with Cy5 fluorophore (excitation 650 nm) at the 5’ end for signal detection. Fresh gonadal tissue from 12-month-old red crucian carp (RCC), white crucian carp (WCC), and their distant hybrid strains (WR and WRII) was embedded in paraffin, and sections were prepared (*n* = 3) and rehydrated in a gradient of xylene, absolute ethanol, 95% ethanol, 80% ethanol, 70% ethanol, and DEPC water. The sections were subsequently treated at room temperature with a 1:9 mixture of 30% H_2_O_2_ and pure methanol to increase probe permeability. To reduce the background, the charged basic proteins in the tissue were neutralized with 0.25% hydrochloric acid. Proteinase K digestion was performed to expose the masked target circRNA, increasing the probe binding efficiency. The enzymatic reaction was terminated via the addition of 0.1 mol/L glycine. Further background reduction was achieved with acetic anhydride, and the tissue was hybridized with prehybridization solution at 65 °C for 1 h, followed by incubation with the designed specific probes for 46 h. Excess probes were washed away using a saline/buffering salt solution, PBS (RNase-free), and DEPC-treated water. After nuclear staining with ProLong Gold Antifade Reagent with DAPI (Invitrogen, Carlsbad, CA, USA), sections were observed under a fluorescence microscope (Leica, Wetzlar, Germany), and Cy5 fluorescence signals were visualized upon excitation at 650 nm, indicating the expression pattern of circdmrt1. β-actin was used as a positive control in the in situ hybridization assay, and a probe against the sense strand of circdmrt1 was designed as a negative control. Image J (1.51j8) software was used to assist in visualizing the expression distribution. The probes are listed in Table S2.

### 4.12. RNA FISH Combined with Immunofluorescence

Fresh gonadal tissue from 12-month-old red crucian carp was embedded in paraffin, and sections were generated. In situ hybridization was performed via an in situ hybridization kit (GeFan, Zug, Switzerland). The circdmrt1 probe was diluted to 1:500. After 48 h, GDF11 (HUABIO, R1510-15, Woburn, MA, USA) and DHRSX antibodies (CUSABIO, PA836670LA01HU, Wuhan, China) were diluted 1:100 in PBS and incubated in the dark at 4 °C for 24 h. Then, the sections were incubated with a fluorescent secondary antibody (APExBIO, K1206, Houston, TX, USA) at room temperature for 30 min, followed by a 5-min wash with PBS. After the nuclei were stained with DAPI, they were observed and photographed under a microscope.

### 4.13. CCK-8 Assay

For HEK-293T cells at 80% density, routine transfection was performed via Lipofectamine 3000 (Invitrogen). The amounts of circdmrt1 used were 1, 2, and 4 µg, and pcDNA 3.1 was used as a blank control. After 24 h, 5000 cells were seeded into a 96-well plate. The mixture was cultured for 6 h, and then 10 µL of CCK-8 (Beyotime C0038, Shanghai, China) solution was added. The mixture was further incubated for 4 h. The absorbance at OD450 nm was measured via a multifunctional microplate reader.

### 4.14. Plasmid Construction and Transfection

The wild-type (WT) and mutant (MUT) target sequences containing the predicted binding sites were synthesized and cloned into the multiple cloning site (MCS) downstream of the firefly luciferase gene in the pGLO luciferase reporter vector (Promega, Madison, WI, USA). The mutant sequence was generated by introducing site-directed mutations within the seed region of the putative miRNA binding site. Successful construction of both wild-type and mutant luciferase plasmids was verified though Sanger sequencing. The regulatory sequences cloned upstream of the luciferase gene consisted of the circdmrt1 fragment and *dhrsx* 3’UTR containing the predicted miR-122-5p binding site (WT) or the corresponding mutated sequence (MUT), both under the control of the SV40 promoter. Detailed sequences of the cloned regulatory regions are provided in Table S3. The Renilla luciferase gene, driven by a constitutive promoter, was included in the same vector and served as the internal control.

HEK-293T cells were maintained in DMEM (Gibco) supplemented with 10% fetal bovine serum (FBS, Gibco) and 1% penicillin-streptomycin (Gibco) at 37 °C with 5% CO_2_. Cells were seeded into 24-well plates at a density of 5 × 10^4^ cells per well 24 h prior to transfection. At approximately 70–80% confluence, cells were co-transfected using Lipofectamine 3000 reagent (Thermo Fisher Scientific, Waltham, MA, USA) according to the instructions. Each well received 500 ng of either the wild-type or mutant luciferase reporter plasmid and 100 nM of miR-122-5p mimics or negative control miRNA (NC mimics, GenePharma, Suzhou, China). Co-transfection was performed in triplicate for each experimental condition. The luciferase activity ratio from each well was first calculated, and the mean value of the three technical replicates was used as a single biological data point for statistical analysis. No-treatment controls (cells without any transfection) were included to establish baseline luminescence for background correction.

### 4.15. Luciferase Gene Reporter Assay

At 48 h post-transfection, cells were harvested and lysed with 1× Passive Lysis Buffer (Promega, Madison, WI, USA) supplied with the Dual-Luciferase Reporter Assay System. Cells were incubated with lysis buffer for 15 min at room temperature with gentle shaking to ensure complete lysis. Luciferase activities were measured using the Dual-Luciferase Reporter Assay System (Promega, Madison, WI, USA) on a GloMax 96 Microplate Luminometer (Promega, Madison, WI, USA). For each sample, 20 μL of lysate was mixed with 50 μL of Luciferase Assay Reagent II (LAR II) to quantify firefly luciferase activity, followed by the addition of 50 μL of Stop & Glo Reagent to measure Renilla luciferase activity. All measurements were performed in technical triplicates for each biological replicate. For each sample, firefly luciferase activity (reporter) was normalized to Renilla luciferase activity (internal control) to account for variations in transfection efficiency and cell viability, and background luminescence was determined using cell lysates from non-transfected cells processed in parallel. For each measurement, the background RLU value was subtracted from all experimental samples. For comparison across independent experiments, normalized luciferase activities were scaled to the control group (negative control mimic + WT construct), which was set to 1.0 (or 100%). This scaling facilitates the comparison of relative changes in reporter activity across replicate experiments. Each sample was assayed in triplicate (technical replicates), and the average value of the three replicates was used to calculate the relative expression level for each biological replicate. All data are presented as the mean ± SD from three independent biological replicates. Statistical significance between groups was determined using Student’s *t*-test, with significance thresholds set as * *p* < 0.05, ** *p* < 0.01, and *** *p* < 0.001.

### 4.16. RNA Immunoprecipitation

HEK-293T cells co-transfected with circdmrt1 and pcDNA-Flag-Ago2 plasmids were collected. Next, 500 µL of 1× lysis buffer was added, and the cells were lysed at −80 °C for 24 h. The next day, 100 µL of protein A/G magnetic beads was removed and washed thoroughly with 300 µL of NT-2 buffer three times. Then, 5 µg of the target antibody and IgG was added, and the mixture was incubated on a rotating shaker at 4 °C for 2 h. The supernatant was discarded, and the beads were washed three times with NT-2 buffer. A total of 400 µL of cell lysate was added to the beads, which were mixed well and then incubated overnight. The next day, the supernatant was discarded, the beads were washed three times with NT-2 buffer, and the proteins were digested with proteinase K at 55 °C for 30 min. The resulting supernatant was used to extract RNA using TRIzol (Invitrogen, Carlsbad, CA, USA)-chloroform (Aladdin Biochemical Technology Co., Ltd, Shanghai, China)-isopropanol (Aladdin Biochemical Technology Co., Ltd, Shanghai, China), followed by reverse transcription into cDNA for subsequent qPCR analysis. For data normalization, the input sample represented 10% of the total cell lysate, whereas the immunoprecipitated (IP) sample was derived from 90% of the lysate. To correct for this 10-fold difference, input Ct values were adjusted using the formula: Ct (Input_adjusted) = Ct (Input_raw) − log_2_ (10) (where log_2_ (10) ≈3.32). The normalized Ct value (ΔCt) for each IP sample was calculated relative to the adjusted input: ΔCt [RIP/Input] = Ct (IP) − Ct (Input_adjusted). Fold enrichment of the target RNA in the specific antibody IP over the IgG control was calculated using the comparative Ct (2^−^ΔΔCt) method: Fold Enrichment = 2^−^ (ΔCt (protein IP) − ΔCt (IgG)). A fold enrichment greater than 2 was considered biologically significant.

### 4.17. Western Blot

Total protein was extracted using RIPA lysis buffer (Beyotime, P0013B, Shanghai, China), separated by SDS-PAGE, transferred to PVDF membranes (Millipore), and blocked with 5% non-fat milk for 2 h. Membranes were incubated with primary antibodies at 1:1000 against FLAG (Proteintech, 20543-1-AP, Rosemont, IL, USA), DHRSX (CUSABIO, PA836670LA01HU, Wuhan, China), and Ago2 (Proteintech, 67934-1-lg, Rosemont, IL, USA), followed by HRP-labeled secondary antibody (Immunoway, RS0002, Plano, TX, USA) at a 1:3000 dilution. Protein bands were visualized and quantified using ImageJ software v1.54t, with target protein signals normalized to GAPDH, and the relative expression of the target protein was determined as the ratio of DHRSX to GAPDH gray values. All experiments were performed with at least three independent replicates, and data are presented as mean ± SD. Statistical comparisons between two groups were analyzed using unpaired two-tailed Student’s *t-*test, while comparisons among three or more groups were performed using one-way ANOVA followed by a post hoc test. Significance was set at * *p* < 0.05, ** *p* < 0.01, and *** *p* < 0.001.

### 4.18. In Vivo Modulation Experiments

For the BSJ locus of the circdmrt1 sequence in RCC, two specific siRNAs were designed, with detailed sequences provided in Table S4. The red crucian carp were cultured in a recirculating water system maintained at 28 °C. Six-month-old male fish (N = 6/group), selected for their uniform body size and weighing 200 ± 20 g, were subjected to intraperitoneal injection at a dosage of 10 μg per fish for three consecutive days. On the fourth day, various tissues (liver, brain, heart, testis) were harvested, and RNA was extracted for qRT-PCR analysis to assess the interference efficiency.

### 4.19. Statistical Analysis

All statistical analyses were performed using GraphPad Prism 9.0 software (GraphPad Software, San Diego, CA, USA). Data normality was assessed using the Shapiro–Wilk test, and homogeneity of variances was evaluated using Levene’s test. For comparisons between two groups, a two-tailed Student’s *t*-test was applied when data met the assumptions of normality; for comparisons among three or more groups, one-way ANOVA followed by Tukey’s post hoc test was used when data met both normality and homogeneity of variances; when data violated the assumptions of normality or homogeneity of variances, the non-parametric Kruskal–Wallis test followed by Dunn’s post hoc test was employed. In this study, for all qPCR experiments, each biological replicate was assayed in technical triplicate, and the mean value was used for subsequent analysis. All graphical data are presented as the mean ± SD derived from biological replicates (*n* = 3).

The threshold for statistical significance was set at *p* < 0.05. Significance markers used in the figure legends are * *p* < 0.05, ** *p* < 0.01, and *** *p* < 0.001, while ns indicates not significant (*p* > 0.05).

## Figures and Tables

**Figure 1:**
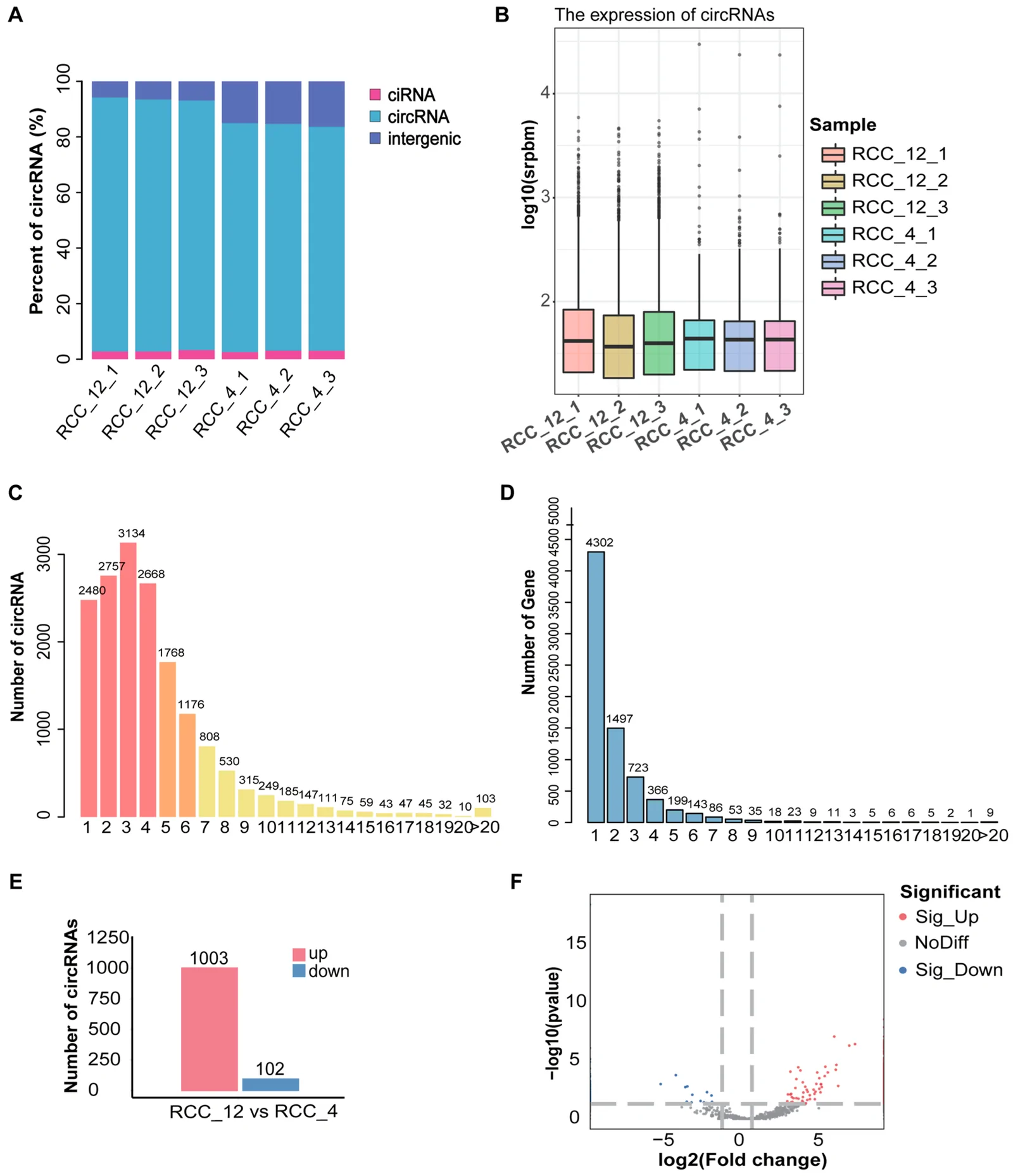
Profiling of circRNAs during testis development in RCC. (**A**) Classification of circRNAs in 4-month-old and 12-month-old RCC. Intron-derived circRNAs (ciRNAs), exon-derived circRNAs (circRNAs), and circRNAs originating from intergenic regions (intergenic). (**B**) Box plot displaying the expression distribution statistics of identified and quantified circRNAs in RCC testes. (**C**) Statistical analysis of the number of exons in the identified circRNAs. The ciRNAs are set to have one exon. (**D**) Distribution of parent genes from which the circRNAs originated. The x-axis represents the number of circRNAs produced by parent genes, and the y-axis indicates the number of parent genes with a specific number of circRNAs. (**E**) The number of differentially expressed circRNAs in the testis at two developmental stages. (**F**) Volcano plot displaying the expression levels of differentially expressed circRNAs.

**Figure 2:**
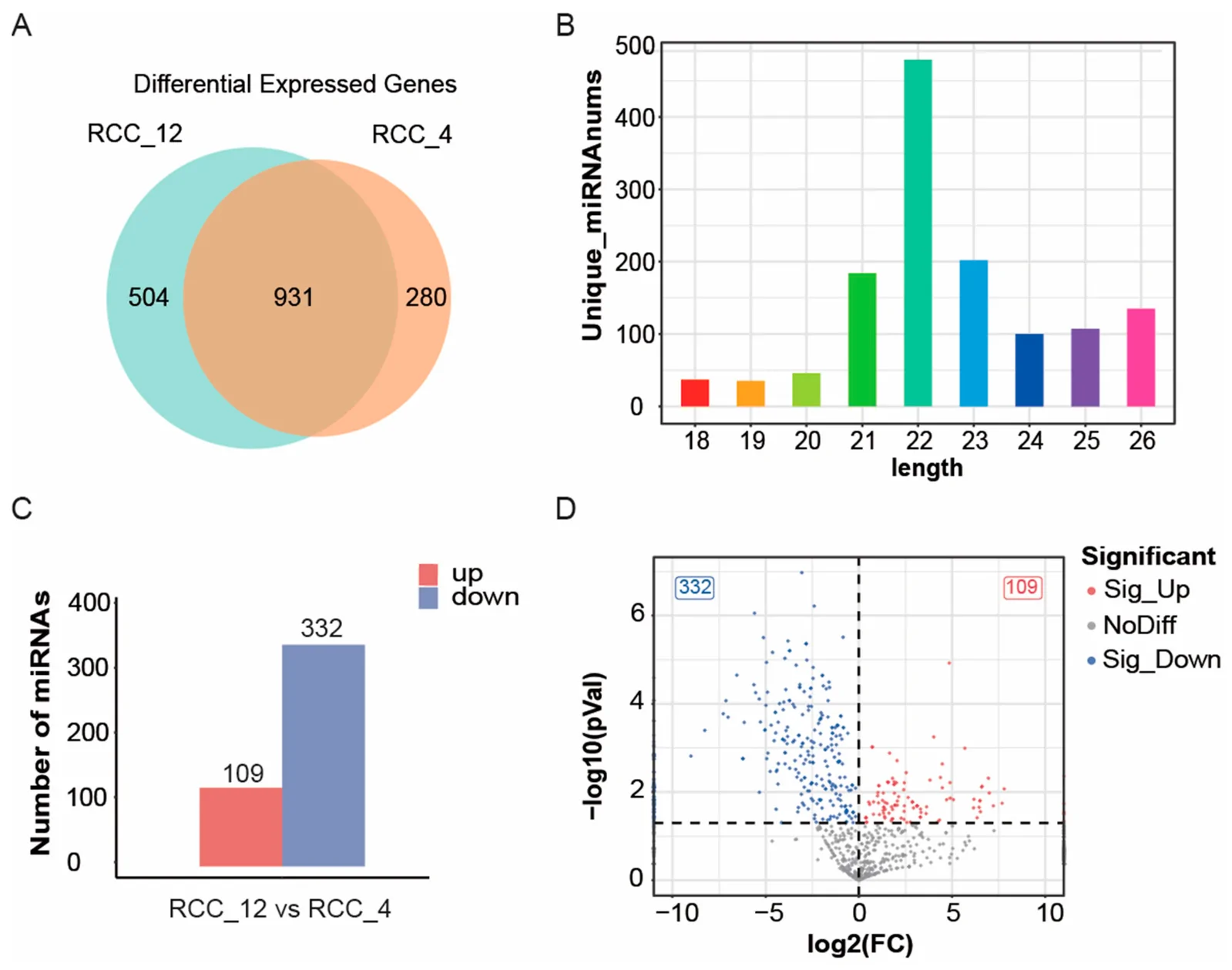
Analysis of miRNAs during testis development. (**A**) Summary of the number of miRNAs at different developmental stages in RCC testes. (**B**) Length distribution statistics of miRNAs. The bars in different colors represent miRNAs of different lengths. (**C**) Bar chart showing the number of differentially expressed miRNAs. (**D**) Volcano plot illustrating the number of differentially expressed miRNAs.

**Figure 3:**
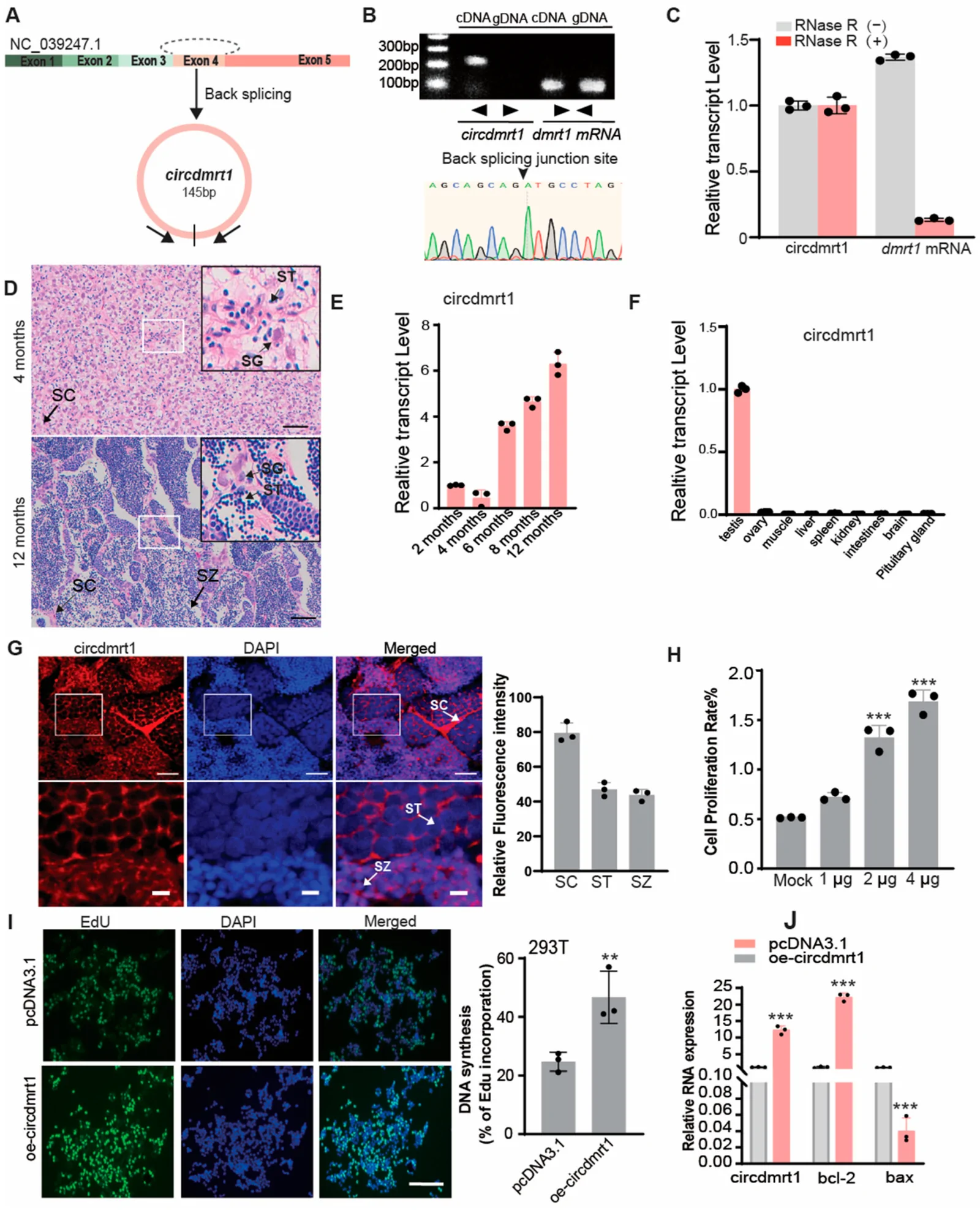
Characterization of circdmrt1 in RCC. (**A**) Genomic locus of circdmrt1 in the dmrt1 gene. circdmrt1 is formed by the back-splicing of the fourth exon of the dmrt1 locus. (**B**) (**Top**) RT-PCR products identified by divergent and convergent primers showing the circularization of circdmrt1 in the testes of 12-month-old RCC. Arrows pointing in opposite directions indicate divergent primers, whereas arrows pointing toward each other indicate convergent primers (as control). (**Bottom**) The back-splicing junction site of circdmrt1 was identified by Sanger sequencing. (**C**) The expression of circdmrt1 and linear dmrt1 mRNA in RCC testes was measured by qRT-PCR in the presence or absence of RNase R. (**D**) HE staining of RCC testes. The upper image shows 4-month-old RCC testes, and the lower image shows 12-month-old testes. Scale bars are 50 μm. (**E**) Quantitative analysis of circdmrt1 expression in the testes of RCC at different ages via qRT-PCR. (**F**) qRT-PCR revealed the relative expression abundance of circdmrt1 in nine RCC tissues. (**G**) RNA fluorescence in situ hybridization of circdmrt1 in RCC testes. The circdmrt1 probe is labeled with Cy5 (red signal). The cell nuclei were stained with 4′,6-diamidino-2-phenylindole (DAPI). The lower row shows an enlargement of the area indicated by the rectangle in the upper row. The right panel shows the fluorescence intensity of circdmrt1. The lower panel is a magnified view of the field within the white box. The scale bars for the upper and lower rows are 20 μm and 5 μm, respectively. (**H**) The viability of HEK-293T cells transfected with pCDNA3.1 or oe-circdmrt1 was assessed using a CCK-8 kit with the indicated amount of plasmid. Mock indicates pCDNA3.1. (**I**) (**Left panel**) DNA synthesis assessed via a 5-ethynyl-2′-deoxyuridine (EdU) assay in HEK-293T cells transfected with the pCDNA3.1 or oe-circdmrt1 plasmid. The cells were fluorescently stained with EdU (green). Nuclei were stained with DAPI (blue). Scale bar, 50 μm. (**Right panel**) Quantitative EdU assay data. (**J**) Overexpression of circdmrt1 attenuated the expression of apoptosis-related genes. HEK-293T cells were transfected with pCDNA3.1 or oe-circdmrt1. The expression of circdmrt1, bcl-2, and bax was determined by qPCR. Data are presented as means ± SDs of three biological replicates (*n* = 3). For RT-qPCR, each biological replicate was assayed in technical triplicate, and the mean value was used for subsequent analysis. (**) *p* < 0.01, (***) *p* < 0.001. Abbreviations: (SC) Sertoli cell, (SG) spermatogonium; (ST) spermatocyte; (SZ) spermatozoa.

**Figure 4:**
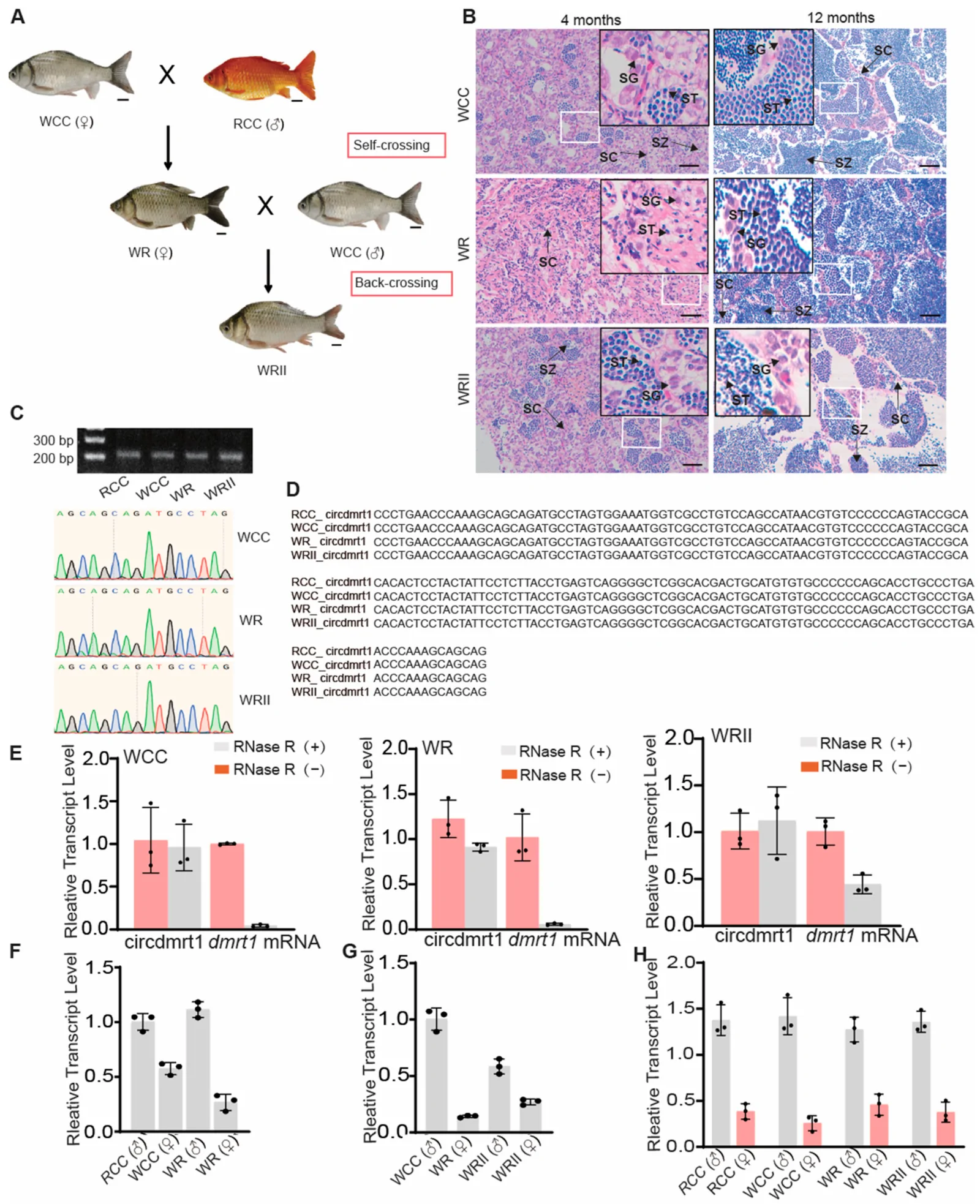
Genetic characteristics of circdmrt1 in WCC, WR, and WRII. (**A**) Crossing procedure for the formation of WR and WRII. Scale bars are 2 cm. (**B**) HE staining of WCC, WR, and WRII; the left image shows a 4-month-old testis, and the right image shows a 12-month-old testis. The black box at the upper left is a magnified view of the white box. Scale bars are 50 μm. (**C**) (Top panel) RT-PCR product of circdmrt1 in WCC, WR, and WRII. (Bottom panel) The back-splicing junction site of circdmrt1 was identified by Sanger sequencing. (**D**) Sequence alignment of circdmrt1 in RCC, WCC, WR, and WRII. (**E**) The expression of circdmrt1 and linear *dmrt1* mRNA in WCC, WR, and WRII testes was detected via qRT-PCR in the presence or absence of RNase R. (**F**) Quantification of circdmrt1 in WR gonads and parental gonads via qRT-PCR. (**G**) Quantification of circdmrt1 in WRII gonads and parental gonads. (**H**) qRT-PCR analysis of the relative abundance of circdmrt1 in the testes and ovaries of RCC, WCC, WR. and WRII. Data are presented as means ± SDs of three biological replicates (*n* = 3). For RT-qPCR, each biological replicate was assayed in technical triplicate, and the mean value was used for subsequent analysis. Abbreviations: (SC) Sertoli cell, (SG) spermatogonium; (ST) spermatocyte; (SZ) spermatozoa.

**Figure 5:**
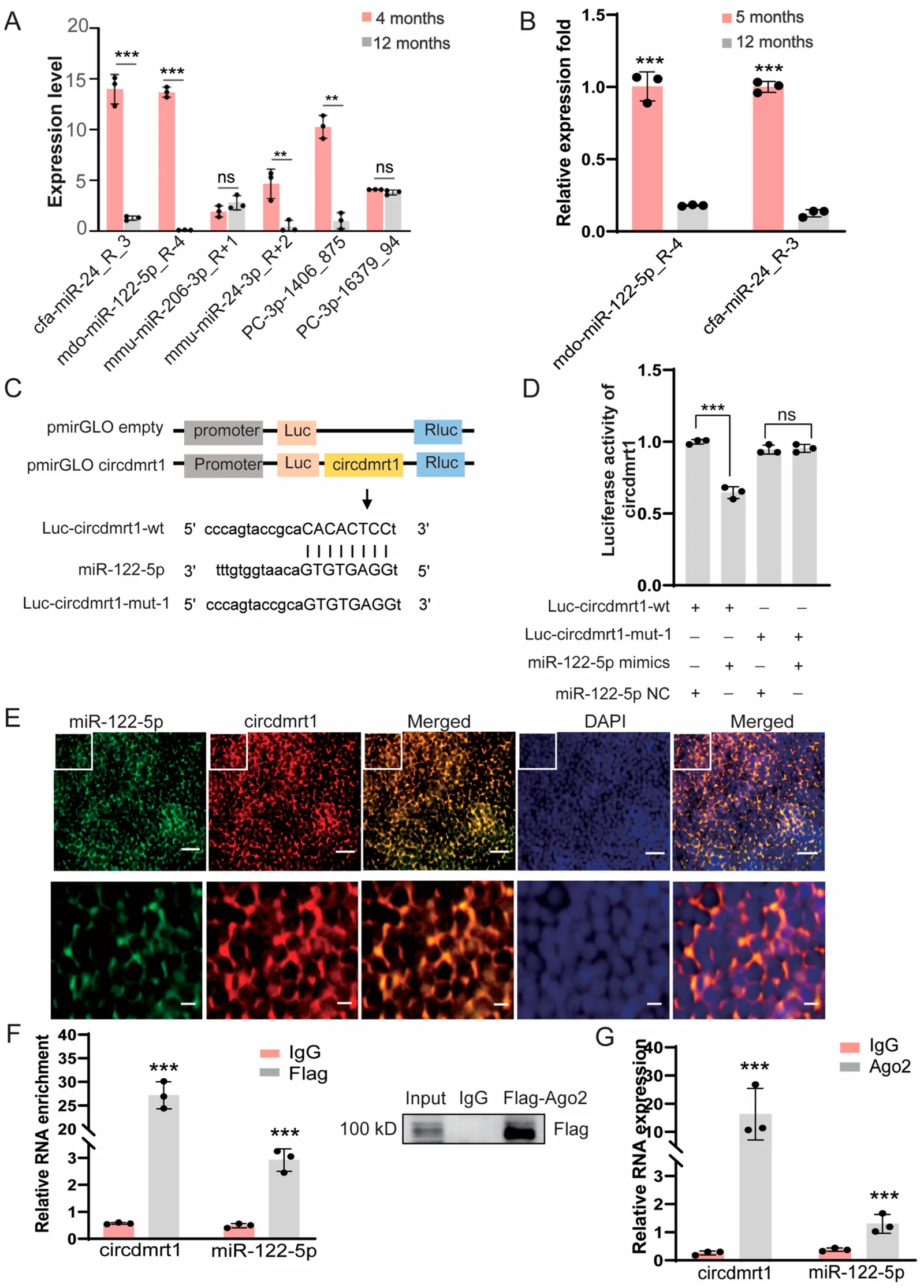
Circdmrt1 physically associates with miR-122-5p. (**A**) The expression abundance of target miRNAs of circdmrt1 at the 4-month and 12-month stages was determined by whole-transcriptome sequencing analysis. (**B**) Relative expression of candidate miRNAs in 5-month-old and 12-month-old RCC testes. (**C**) Putative binding sites of miR-122-5p on circdmrt1. (**D**) Luciferase activity of circdmrt1 in HEK-293T cells transfected with miR-122-5p mimics. The luciferase activity was normalized to the Renilla luciferase activity of circdmrt1. (**E**) Representative images of RCC testes stained with a miR-122-5p probe (green), a circdmrt1 probe (red), and DAPI (blue). The lower panel is a magnified view of the field within the white box. The scale bars for the upper and lower rows are 20 μm and 5 μm, respectively. (**F**) Ago2 immunoprecipitation was performed in HEK-293T cells transfected with circdmrt1 and miR-122-5p mimics, followed by qRT-PCR and Western blotting to detect circdmrt1 and miR-122-5p transcript levels (**left**) and flag protein levels (**right**), respectively. (**G**) Ago2 immunoprecipitation was performed in RCC testes, followed by qRT-PCR to detect circdmrt1 and miR-122-5p expression. Data are presented as means ± SDs of three biological replicates (*n* = 3). For RT-qPCR, each biological replicate was assayed in technical triplicate, and the mean value was used for subsequent analysis. ns: No significance, (**) *p* < 0.01, (***) *p* < 0.001, two-tailed *t*-test.

**Figure 6:**
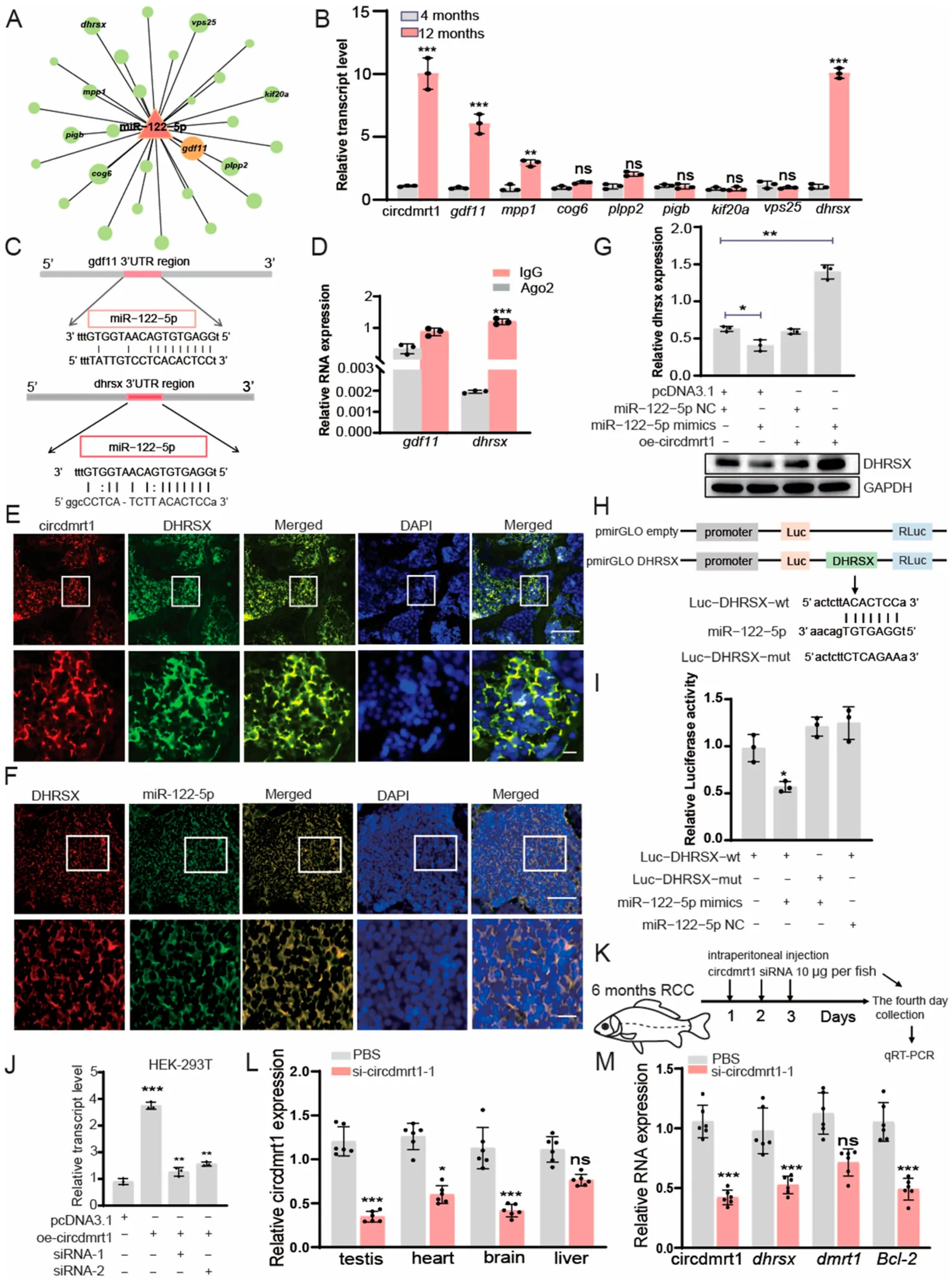
Circdmrt1 relieves the repressive effect of miR-122-5p on dhrsx mRNA and protein expression. (**A**) Diagram of the miRNA miR-122-5p target gene prediction network. (**B**) mRNA levels of putative target genes of miR-122-5p in RCC testes at different developmental stages. (**C**) Putative binding sites of miR-122-5p on GDF11 and DHRSX. (**D**) Ago2 immunoprecipitation was performed in RCC testes, followed by qRT-PCR to detect gdf11 and dhrsx expression. (**E**) Representative images of RCC testes stained with an anti-DHRSX antibody (green), a circdmrt1 probe (red), and DAPI (blue). The scale bars for the upper and lower rows are 25 μm and 5 μm, respectively. (**F**) Representative images of RCC testes stained with an anti-DHRSX antibody (red), an miR-122-5p probe (green), and DAPI (blue). The scale bars for the upper and lower rows are 25 μm and 10 μm, respectively. The lower panel is a magnified view of the field within the white box. (**G**) Detection of DHRSX transcript and protein levels via qRT-PCR and Western blotting in HEK-293T cells transfected with miR-122-5p mimics and oe-circdmrt1. (**H**) Putative binding sites of miR-122-5p on DHRSX. (**I**) Luciferase activity of DHRSX in HEK-293T cells transfected with miR-122-5p mimics, normalized to the Renilla luciferase activity of DHRSX. (**J**) Expression levels of circdmrt1 in HEK-293T cells transfected with oe-circdmrt1 and treated with circdmrt1 siRNA. (**K**) Schematic diagram of the siRNA injection in RCC (*n* = 6). (**L**) qRT-PCR analysis of circdmrt1 expression levels in the testis, heart, liver, and brain of RCC. (**M**) qRT-PCR analysis of the expression levels of circdmrt1, *dmrt1* mRNA, *dhrsx*, and *bcl-2* in the testis of RCC. Data are presented as means ± SDs of six biological replicates (*n* = 6). For qRT-PCR, each biological replicate was assayed in technical triplicate, and the mean value was used for subsequent analysis. Ns: No significance, (∗) *p* < 0.05, (∗∗) *p* < 0.01, (∗∗∗) *p* < 0.001, two-tailed *t*-test.

**Figure 7:**
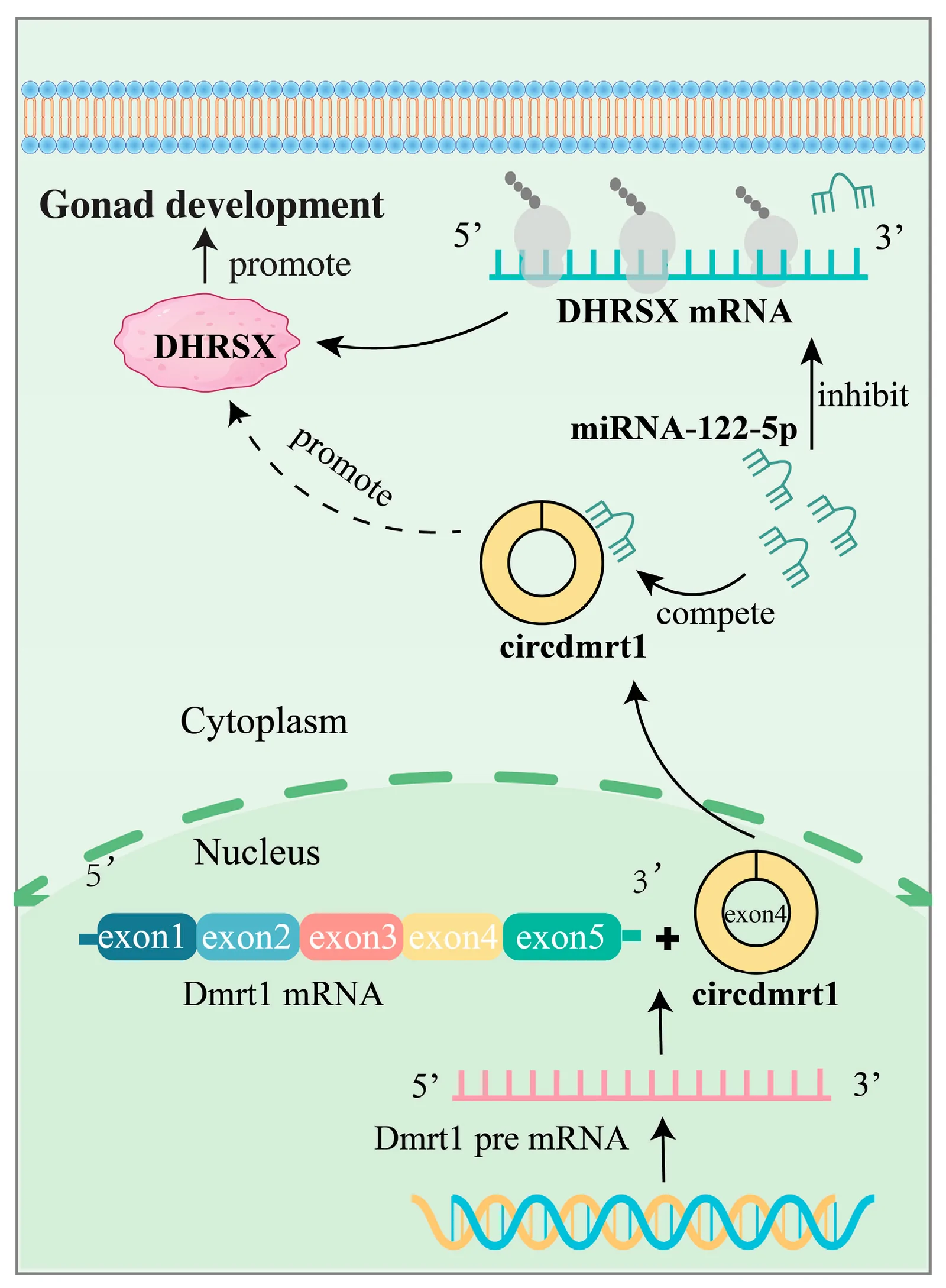
Diagram illustrating the hypothetical mechanism of circdmrt1/miR-122-5p/DHRSX crosstalk during testis development in crucian carp. In crucian carp, circdmrt1 exhibited increased testis-specific expression during the developmental stage, may act as a miRNA sponge for miR-122-5p, thereby increasing the expression of DHRSX. Thus, the expression of antiapoptotic genes and proapoptotic genes is altered, and cell proliferation is promoted, triggering male development.

## Data Availability

Sequencing data have been deposited in the National Center for Biotechnology Information (NCBI) under the Sequence Read Archive (SRA) accession code PRJNA1313861 (27 April 2026) (https://www.ncbi.nlm.nih.gov/bioproject/PRJNA1313861). All other relevant data and details of resources can be found within the article and its Supplementary Information.

## Supplementary Materials

The following supporting information can be downloaded at https://www.mdpi.com/article/10.3390/ijms27198593/s1.

## Abbreviations

The following abbreviations are used in this manuscript:

RCC	Red crucian carp
WCC	White crucian carp
Ago2	Argonaut 2
CircRNA	Circular RNA
ceRNA	Competitive endogenous RNA
