# Supplementary material for: Comprehensive circRNA Profiling Reveals a Testis-Specific circRNA circdmrt1 as a Novel Regulator of Gonadal Development in Crucian Carp
Source: Int J Mol Sci. 2026 Sep 25;27(19):8593. doi: 10.3390/ijms27198593 (PMC13664762; doi:10.3390/ijms27198593)
Supplement: Supplementary file 1 [file ijms-27-08593-s001.zip › ijms-4514211-supplementary.pdf]

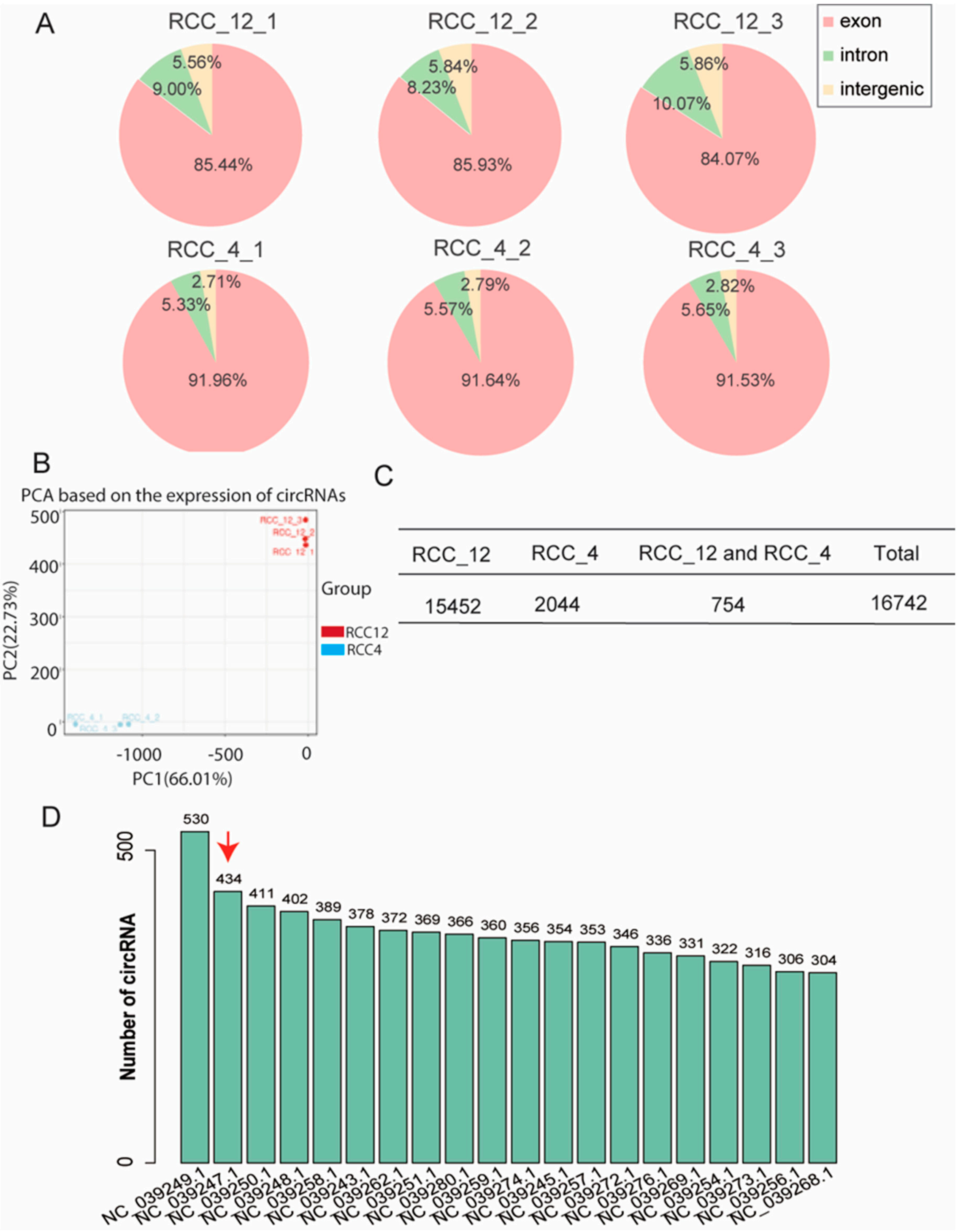

Figure S1. Profiling of circRNAs in RCC. (A) The distribution of reads in testes from RCCs of different ages that did not align with the reference genome was analyzed via TopHat-Fusion for nonlinear alignment. The reads were mapped to exons, introns, and intergenic regions. (RCC_12_1) refers to the first transcript from a 12-month-old red crucian carp testis, and (RCC_4_1) refers to the first transcript from a 4-month-old red crucian carp testis, with three transcripts representing three replicates. (B) Principal component analysis (PCA). (C) Statistics of circRNA numbers. (D) Chromosomal distribution of circRNA origins, with arrows indicating the chromosome containing the gene doublesex and mab-3-related transcription factor 1 (dmrt1), which encodes circdmrt1.

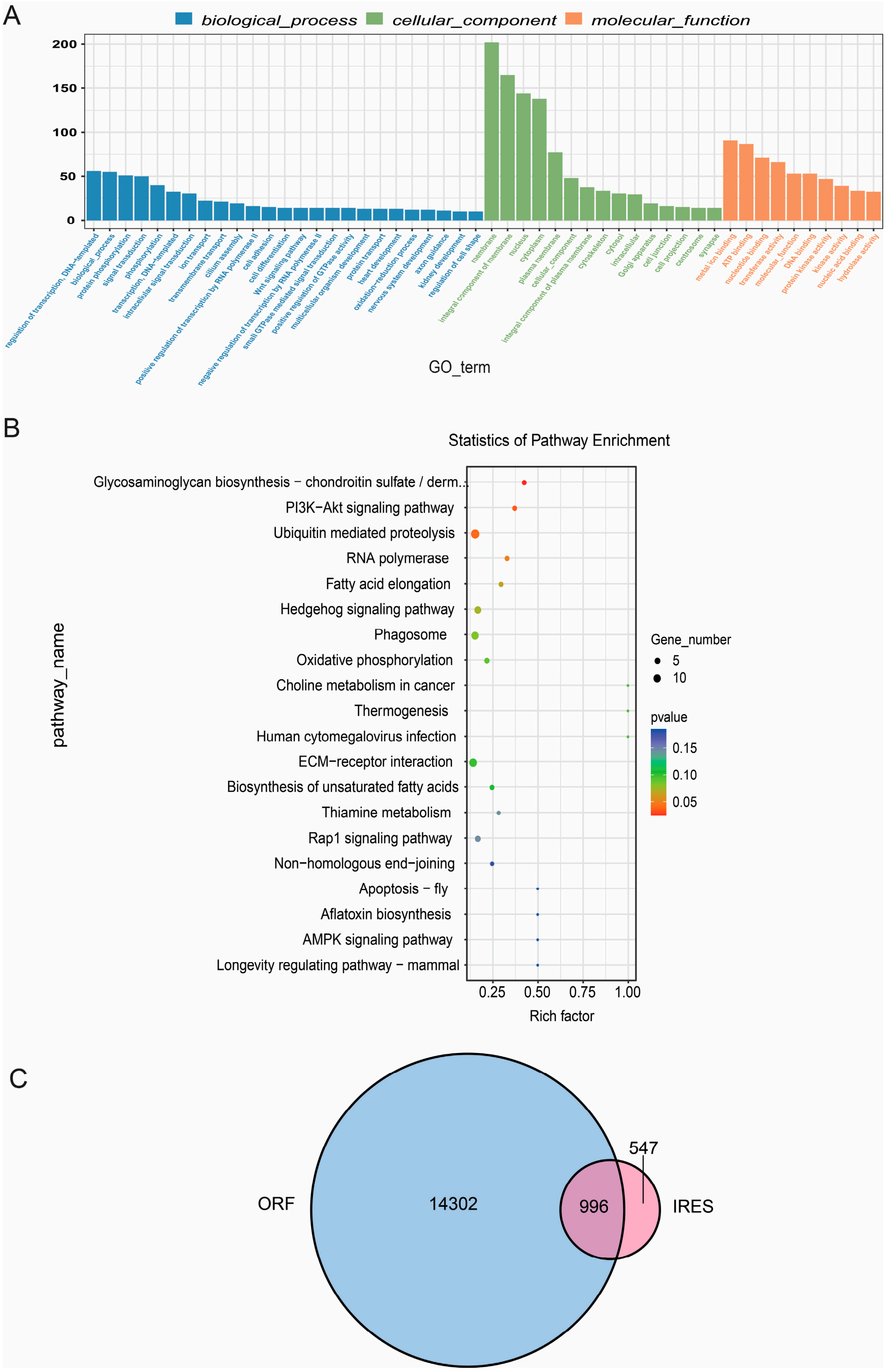

Figure S2. GO and KEGG analyses of circRNAs in red crucian carp. (A) GO enrichment analysis of significantly differentially expressed circRNAs. (B) KEGG enrichment analysis of circRNAs. (C) Prediction of the number of circRNAs with ORF and IRES sites in the testis of RCC.

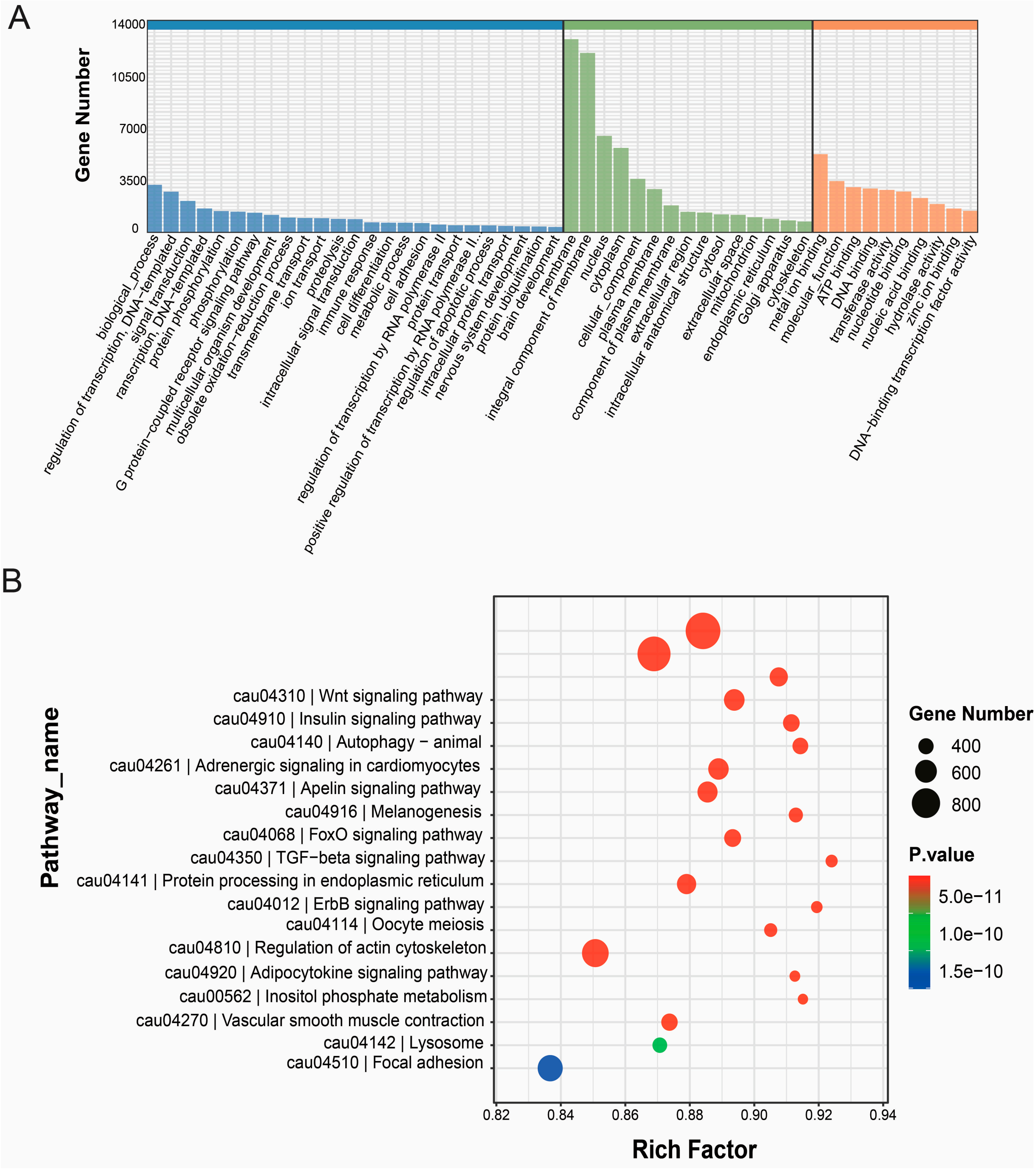

Figure S3. GO and KEGG analyses of microRNAs in RCC. (A) GO enrichment analysis of the target genes of the differentially expressed miRNAs. (B) KEGG enrichment analysis of target genes for differentially expressed miRNAs.

A

Accession	chrom	start	end	strand	exon Count	circType	isoform Name	gene Name
circRNA6228	NC_039247.1	17333357	17333501	+	1	circRNA	XM_026251216.1	LOC113079027

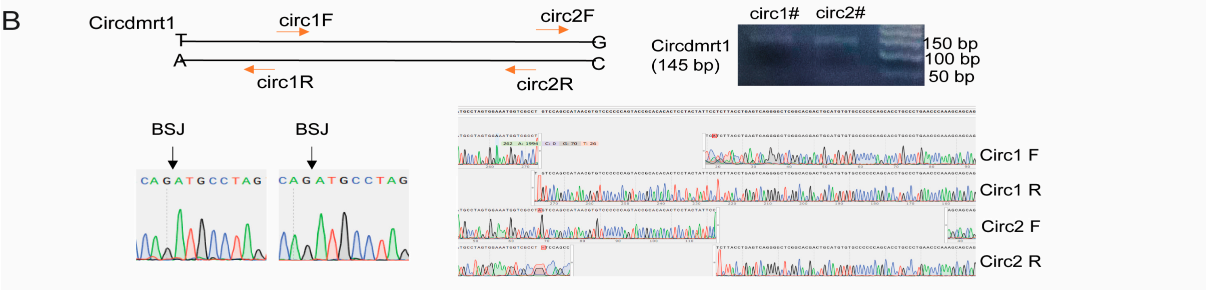

Figure S4. Validation of the circdmrt1 sequence. (A) Classification and distribution of circRNAs in the RCC genome. (B) Full-length circdmrt1 was amplified by PCR using BSJ-spanning primers and confirmed by Sanger sequencing.

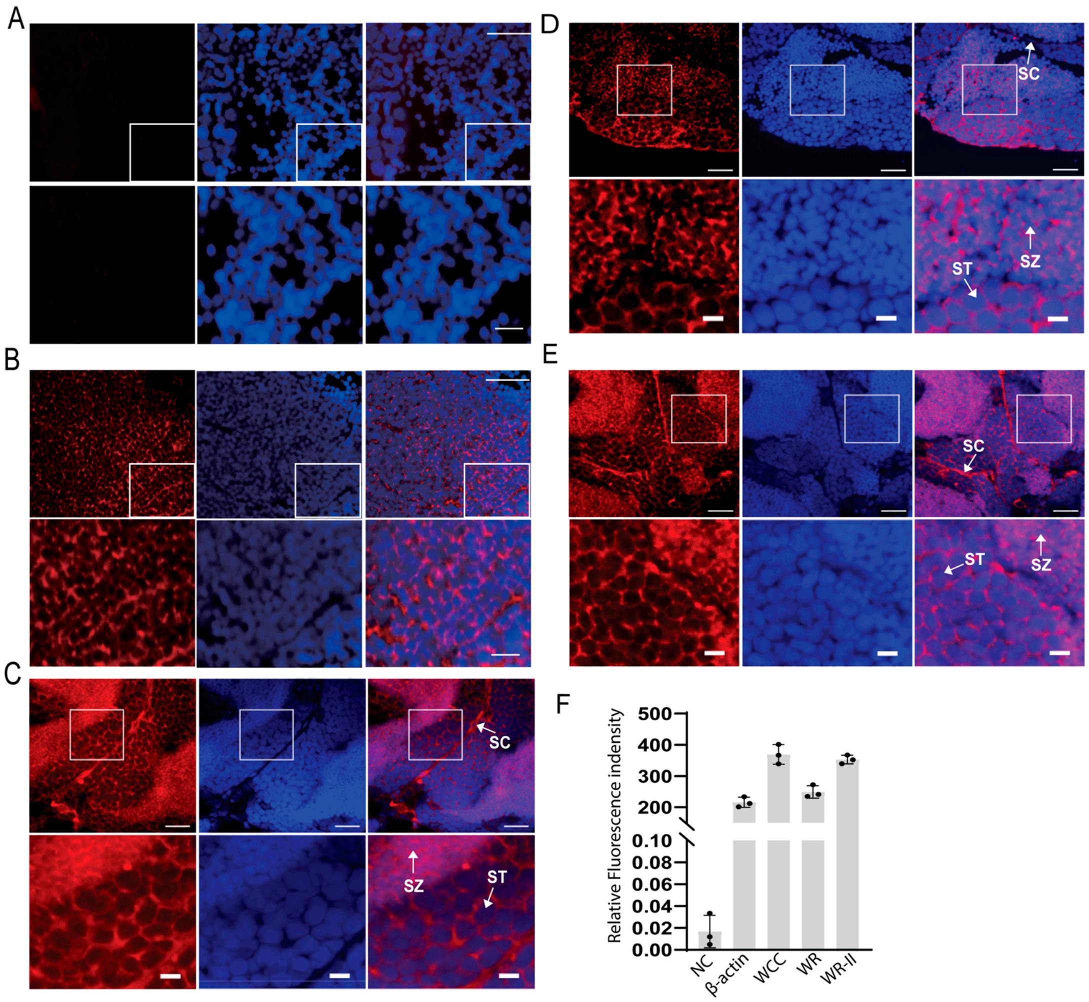

Figure S5. RNA fluorescence in situ hybridization study of circdmrt1 in WCC, WR and WRII. (A, B) RNA fluorescence in situ hybridization of β-actin (B) and the sense strand of circdmrt1 (C) in RCC. The probe is labeled with Cy5 (red signal). The cell nuclei were stained with 4',6-diamidino-2-phenylindole (DAPI). (C-E) RNA fluorescence in situ hybridization of circdmrt1 in WCCs (C), WRs (D) and WRIIs (E). The circdmrt1 probe is labeled with Cy5 (red signal). The cell nuclei were stained with 4',6-diamidino-2-phenylindole (DAPI). The lower row shows an enlargement of the area indicated by the rectangle in the upper row. The scale bars for the upper and lower rows are 20 μm and 5 μm, respectively. (F) Bar chart for fluorescence quantitative analysis.

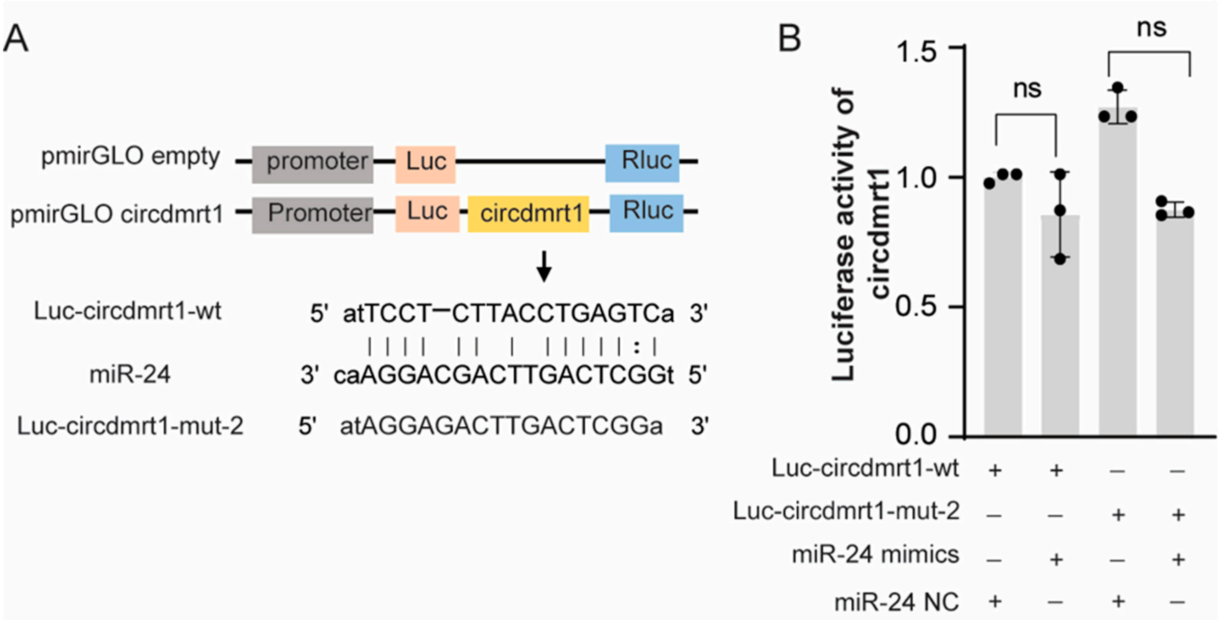

Figure S6. Circdmrt1 doesn't associates with miR-24. (A) Putative binding sites of miR-24 on circdmrt1. (B) Luciferase activity of circdmrt1 in HEK293T cells transfected with miR-24 mimics. The luciferase activity was normalized to the Renilla luciferase activity of circdmrt1.

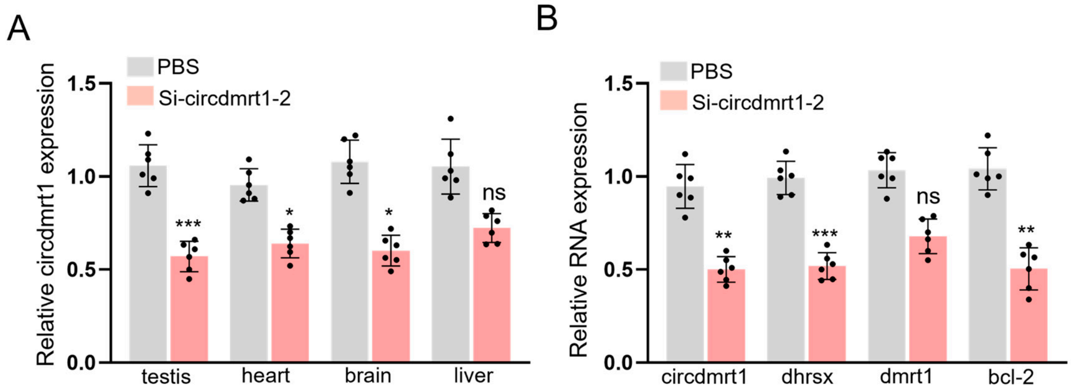

Figure S7. The knockdown effect of in vivo injection of si-circdmrt1-2 in crucian carp. (A) qRT-PCR analysis of circdmrt1 expression levels in the testis, heart, liver, and brain of RCC. (B) qRT-PCR analysis of the expression levels of circdmrt1, dmrt1 mRNA, dhrsx, and bcl-2 in the testis of RCC, Data are presented as means ± SDs of three biological replicates (n = 6). For RT-qPCR, each bio-logical replicate was assayed in technical triplicate, and the mean value was used for subsequent analysis.

Table S1. RT-PCR Primer information in this study

Name	Species	Sequence (3'-5')
circdmrt1-F	crucian carp	ACGACTGCATGTGTGCC
circdmrt1-R	crucian carp	ATTTCCACTAGGCATCTGCTG
Dmrt1-F	crucian carp	TAGTGGAAATGGTCGCCTGT
Dmrt1-R	crucian carp	TGACTCAGGTAAGAGGAATAGTAGG
Circdmrt1-F-1	crucian carp	GTCCAGCCATAACGTGTCC
Circdmrt1-R-1	crucian carp	AGGCGACCATTTCCACTAGG
Circdmrt1-F-2	crucian carp	TCTTACCTGAGTCAGGGGCT
Circdmrt1-R-2	crucian carp	GGAATAGTAGGAGTGTGTGCG
Bcl-2-F	Human	CTGCTGTGGCTTCTGTGGTCTTG
Bcl-2-R	Human	CACTTCTGTGTCAGGTTCCCTTGTC
BAX-F	Human	ATTTTGCGGGGCGGCCACGT
BAX-R	Human	TGGCAGCGCCCGATCCGGGA
Bcl-2-F	crucian carp	ATGGCACAGGATAATGTGTAC
Bcl-2-R	crucian carp	AAGCCGCTCCAGTTCGTC
BAX-F	crucian carp	CAGCGCCGTCGGGTCCAGGC
BAX-R	crucian carp	GCAAACACTGCGCAAGACGTT
mdo-miR-122-5p-F	crucian carp	GCTGGAGTGTGACAATGGTGTTT
cfa-miR-24-F	crucian carp	CTGGCTCAGTTCAGCAGGAAC
β-actin-F	crucian carp	CATCTACGAGGGTTACGCCC
β-actin-R	crucian carp	AATTTCCCTCTCGGCTGTGG
18s RNA-F	crucian carp	GAGTATGGTTGCAAAGCTGAAAC
18s RNA-R	crucian carp	AATCTGTCAATCCTTTCCGTGTCC
GDF11-F	crucian carp	GCACCTACAAGTCTCTGCAA
GDF11-R	crucian carp	TCCTGGATCTAAGAGTGCTGT
DHRSX-F	crucian carp	TACTCCCCGTAAATCTGCATC
DHRSX-R	crucian carp	GTTCGATAGTTCCGCCTTCT
pip4k2a-F	crucian carp	TGTGTGGACAGACAGCTGAA
pip4k2a-R	crucian carp	CTGTCAAGTCGCTGCAAAAC
cog6-F	crucian carp	TATCGCTGGGCTCAGAATGA
cog6-R	crucian carp	GAACAGGACGATCCTGGAGA
mpp1-F	crucian carp	GACGCTGAAAGTGCTCAGAA
mpp1-R	crucian carp	GGCAGTGTTTGTAGGAGCAA
plpp2-F	crucian carp	CCCCTCTCTGTCCAGATGTT
plpp2-R	crucian carp	GTTTCTGTCCGTGTCTGCAA
pigb-F	crucian carp	GTTTAAACCCCGTCTGAGGC
pigb-R	crucian carp	TCACTGATTCACACGAAGCC
kif20a-F	crucian carp	GGTGTGCAGTTCTCCATCTG
kif20a-R	crucian carp	TGGGACTGCAGTGAAGGTAA
vps25-F	crucian carp	CCAAAAACGGCATGGTCAAC
vps25-R	crucian carp	ATCCTCCAGACCGTGAAACT
GAPDH-F	Human	ACCTTCACTCCTCCATCTT
GAPDH-R	Human	AGGTCACAGACACGGTTG

Table S2. Probe sequences used in the article

Name	Sequence	Purpose
circdmrt1	5' CY5-ATTTCCACTAGGCATCTGCTGCTTTGGG 3'	FISH
mdo-miR-122-5p	5' FAM-AAACACCATTGTCACACTCCA 3'	FISH
Sense-circdmrt1	5' CY5- CCCAAAGCAGCAGATGCCTAGTGGAAAT 3'	FISH
β-actin	5' CY5-GCCTTGACCTCTCTGGCTTTGGGTTGAGGG 3'	FISH

Table S3. Detailed sequences of the cloned regulatory regions

Plasmid	Sense	Purpose
LUC-DHRSX-WT	5’-actcttACACTCCa-3’	Luciferase gene reporter assay
LUC-DHESX-MUT	5’-actcttCTCAGAAa-3’	Luciferase gene reporter assay
LUC-circdmrt1-WT	5’- cccagtaccgcaCACACTCCt-3’	Luciferase gene reporter assay
LUC-circdmrt1-MUT	5’- cccagtaccgcaGTGTGAGGt-3’	Luciferase gene reporter assay
MiR-122-5p minics	3’- aacagTGTGAGGt-5’	Luciferase gene reporter assay

Table S4. Gene-specific siRNA oligo sequence mentioned in the study

Gene	Sense	Antisense
siNC	5’-UUCUCCGAACGUGUCACGUTT-3’	5’-ACGUGACACGUUCGGAGAATT-3’
circdmrt1 siRNA-1	5’- CCCAAAGCAGCAGAUGCCUTT-3’	5’- AGGCAUCUGCUGCUUUGGGTT-3’
circdmrt1 siRNA-2	5’- AAGCAGCAGAUGCCUAGUGTT-3’	5’- CACUAGGCAUCUGCUGCUUTT-3’
